# Development of 3D spheroids of AGS gastric cancer cells *in vitro* for exploration of *H. pylori* influence on tumor environment and anti-cancer activity of PLA-His-DOX nanoparticles

**DOI:** 10.3389/fmicb.2026.1935725

**Published:** 2026-08-24

**Authors:** Patrycja Jaroniek, Magdalena Chmiela, Marek Brzeziński, Weronika Gonciarz

**Affiliations:** 1 Department of Immunology and Infectious Biology, Institute of Microbiology, Biotechnology and Immunology, Faculty of Biology and Environmental Protection, University of Lodz, Łódź, Poland; 2 BioMedChem Doctoral School of the University of Lodz and Lodz Institutes of the Polish Academy of Sciences, University of Lodz, Łódź, Poland; 3 Centre of Molecular and Macromolecular Studies, Polish Academy of Sciences, Łódź, Poland

**Keywords:** drug delivery, gastric cancer, *H. pylori*, nanoparticles, spheroids

## Abstract

**Introduction:**

Gastric cancer (GC) is a complex disease, which may have infectious origin. *Helicobacter pylori* (*H. pylori*) Gram-negative bacteria are linked with GC development. There is growing interest in nanotechnology to develop therapeutic solutions for the treatment of GC *in situ*. The predictive assessment of nanomedicine efficacy in GC remains limited due to the lack of physiologically relevant models that capture tumor architecture and infection-driven reprogramming of gastric environment.

**Methods:**

The aim of this study was to develop 3D culture of GC cell spheroids to assess the impact of selected *H. pylori* components toward this microenvironment. A further goal was to develop polylactic (PLA) nanoparticles (NPs) loaded with anti-cancer drug doxorubicin (DOX) and modified with histamine (His) to improve cellular penetration of the studied NPs and test effectiveness of developed formulation in 3D spheroidal model.

**Results and Disscusion:**

It has been shown that *H. pylori* components, cytotoxin associated gene A (CagA) protein and lipopolysaccharide (LPS) influenced spheroid architecture particularly by enlarging rim zone. PLA-His-DOX NPs induced cytotoxicity of spheroidal GC cells through oxidative stress amplification, DNA damage, and apoptosis. Treatment of GC spheroids with PLA-His-DOX reduced production of pro-inflammatory interleukin (IL) IL-1β and IL-8 driven by *H. pylori* soluble components, indicating a combined cytotoxic and anti-inflammatory mechanism of PLA-His-DOX. *In vivo* study using *Cavia porcellus* confirmed short-term tolerability of the studied unloaded carrier (PLA-His-NPs) under tested conditions with no detectable organ toxicity. In summary, 3D spheroids of AGS GC cells developed in this study *in vitro* consist of predictive models that link tumor biology with infectious components and facilitate the assessment of anti-GC cell effects of PLA-His-DOX NPs. Soluble *H. pylori* CagA and LPS, which *in vivo* can be released from damaged bacterial cells facilitated creating controlled environment in AGS cell cultures *in vitro*. However, this model did not mimic complexity of live bacterial infection. This study prompts further research for deepening the mechanisms of anti-cancer activity and efficacy of the developed PLA-His-NPs.

## 1 Introduction

GC remains a major global health burden characterized by high mortality, heterogeneity, and a strong association with chronic *H. pylori* infection (IARC, 2025; Ferlay et al., 2024; Reyes, 2023), which reprograms gastric microenvironment through sustained inflammatory signaling, disruption of epithelial barrier, and immune modulation, thereby promoting tumor progression and resistance to therapy (Duan et al., 2025; Salvatori et al., 2023). Key virulence factors of *H. pylori*, including cytotoxin associated gene A (CagA) protein and lipopolysaccharide (LPS), drive nuclear factor kappa B (NF-κB) activation and the release of pro-inflammatory cytokines such as interleukin (IL)-1β and IL-8, and remodeling extracellular matrix collectively creating a tumor-permissive and therapy-resistant niche (Crabtree et al., 1994; Kitadai et al., 2000; Mnich et al., 2015). Clinical GC outcomes remain poor due to late diagnosis, multidrug resistance, and profound intratumoral heterogeneity (Guan et al., 2023; Emran et al., 2022). Experimental exposure of GC cells *in vitro* to isolated bacterial components allows studying specific host-cell responses under controlled conditions. However, soluble *H. pylori* CagA and LPS which can be released during infection from damaged bacterial cells do not mimic infection with live bacteria. Therefore, the approach used in this study is regarded as an *H. pylori* component-conditioned model rather than a model of active or chronic *H. pylori* infection. Consequently, this model is not suitable for testing the effects of CagA translocation via type IV secretion system.

Nanomedicine is promising as therapeutic weapon among anti-cancer therapies (Tracey et al., 2021). In addition to conventional polymeric drug-loading strategies, co-assembly approaches involving biologically active compounds illustrate how nanoscale organization may improve cargo stability, bioavailability and pharmacological performance (Miao et al., 2025). However, a major limitation in preclinical research is the reliance on two-dimensional (2D) culture models that fail to capture the spatial, biochemical, and mechanical complexity of solid tumors, including diffusion barriers, oxygen gradients, and extracellular matrix-mediated signaling (Mangani et al., 2025; Arora et al., 2024). These cell models are poorly predictive of NPs transport, penetration, and therapeutic response. NPs-like drug delivery systems facilitate overcoming these barriers by enabling controlled drug release, improved accumulation of therapeutic substance and enhanced intracellular distribution often mediated by the enhanced permeability and retention (EPR) effects (Ioele et al., 2022; Chen et al., 2024). Three-dimensional (3D) cell spheroids more closely replicate avascular tumor architecture, proliferative gradients, hypoxic cores, and conditions of diffusion-limited drug penetration, while maintaining therapy-resistant and invasion-associated phenotypes (Arora et al., 2024; Živković and Opačak-Bernardi, 2025; Achilli et al., 2012; Coelho et al., 2024). In 3D cell cultures, transport primarily relies on diffusion, spheroid size and compactness, cell–cell interactions, and the physicochemical properties of the tested formulation (Ioele et al., 2022; Chen et al., 2024). They may therefore offer a more informative platform for evaluating anticancer formulations than traditional monolayer cultures, although they do not mimic vascular, stromal, and immune components of the tumor microenvironment.

Previously using monolayers of AGS GC cells we demonstrated that polylactide (PLA) nanoparticles (NPs) functionalized with histamine (His) and loaded with doxorubicin (DOX) - PLA-His-DOX showed stronger cytotoxic effect against AGS GC cells than the corresponding soluble compounds: His, DOX, His-DOX or blank PLA NPs or PLA-His NPs (Brzeziński et al., 2023; Jaroniek et al., 2025). This finding prompt to develop further study using 3D GC model to deepen the mechanisms of PLA-His-DOX formulation against GC cells.

Therefore, in this study, a 3D model of AGS GC cells has been established to assess how exposure of cells to soluble *H. pylori* components, CagA and LPS will influence spheroid morphology in conjunction with cell metabolic activity and production of selected cytokines involved in the development of tumor environment. It has been shown that PLA-His-DOX NPs can induce oxidative stress, DNA damage, and apoptosis in conventional AGS cell cultures *in vitro* (Jaroniek et al., 2025).

Preliminary data have suggested that functionalization of PLA NPs with His may facilitate DOX distribution in such cell cultures potentially via interaction of NPs with His receptors, H2 and H4. Furthermore, cell exposure to *H. pylori* CagA or LPS resulted with increased deposition of these receptors on AGS cells (Jaroniek et al., 2026). However, direct receptor-dependent uptake, the contribution of specific endocytic pathways, and the intracellular fate of the carrier have not yet been conclusively established.

In this study we employed PLA-His-DOX NPs and 3D cultures of GC AGS cells to assess: (i) the distribution of DOX delivered by PLA-His-DOX NPs within spheroids of GC cells AGS in conjunction with these cell viability; (ii) the influence of soluble *H. pylori* CagA, *H. pylori* LPS or standard *Escherichia coli* LPS on spheroid morphology, (iii) anti-GC cell activity of DOX and PLA-His-DOX in an *H. pylori* component-conditioned environment; (iv) production of proinflammatory cytokines: interleukin-IL-1β and IL-8 under experimental conditions.

In addition, the aim of this study was to assess a short-term tolerability of blank PLA-His NPs in *Cavia porcellus* model based on selected systemic biochemical and inflammatory markers in serum samples and histological examination of liver, kidney, and spleen tissue sections (preliminary study). We expect that 3D GC cell model will facilitate deepening previous findings on the effects of PLA-His-DOX toward AGS GC cells derived from simplified 2D cell model.

## 2 Materials and methods

### 2.1 Preparation and physicochemical characterization of nanoparticles by nanoprecipitation

Polylactide with histamine end groups (PLA-His) was synthesized as described previously (Brzeziński et al., 2023). Blank PLA-His NPs and DOX-loaded PLA-His NPs (PLA-His-DOX) were prepared by nanoprecipitation according to previously published procedures (Brzeziński et al., 2023; Jaroniek et al., 2025). Briefly, 10 mg of PLA-His and 4 mg of poly (ethylene glycol) methyl ether-block-poly (D,L-lactide) (PEGME-b-PDLLA) were dissolved in 4 mL of tetrahydrofuran. For the preparation of PLA-His-DOX NPs, 300 μL of a 5 mg/mL DOX solution in tetrahydrofuran, corresponding to an initial DOX amount of 1.5 mg, was added to the polymer solution. The organic phase was gradually introduced into 50 mL of distilled water under gentle magnetic stirring. The resulting nanoparticle suspension was filtered through a 0.4 μm polyamide filter, and tetrahydrofuran was removed under reduced pressure. Blank PLA-His NPs were prepared using the same procedure without the addition of DOX. To provide formulation-specific physicochemical data, a newly prepared batch of blank PLA-His and PLA-His-DOX NPs was characterized. Hydrodynamic diameter and polydispersity index were determined by dynamic light scattering, whereas surface charge was assessed by zeta-potential measurements.

The mean hydrodynamic diameter was 67 nm for blank PLA-His nanoparticles and 78 nm for PLA-His-DOX nanoparticles. The corresponding polydispersity index values were 0.236 and 0.184, respectively, and the zeta potentials were −16.0 and −16.8 mV. The physicochemical characteristics are summarized in Supplementary Table 1, and representative particle-size and zeta-potential distributions are provided in Supplementary Figure 1. The physicochemical measurements were performed using a newly prepared batch obtained according to the same nanoprecipitation procedure as that used to prepare the NPs for the biological experiments.

### 2.2 DOX encapsulation efficiency, nominal drug loading and concentrations used in biological experiments

The encapsulation efficiency of DOX was determined after lyophilization of the DOX-loaded NPs and their dissolution in dimethyl sulfoxide, as described previously (Brzeziński et al., 2023). Fluorescence intensity was measured at an excitation wavelength of 490 nm and an emission wavelength of 560 nm, and the amount of encapsulated DOX was determined using a calibration curve. The encapsulation efficiency of the newly prepared PLA-His-DOX formulation was 49%. Based on the initial DOX amount of 1.5 mg, the calculated amount of encapsulated DOX was 0.735 mg. The nominal drug loading was calculated according to the following equation: Drug loading (%) = [mass of encapsulated DOX/(total polymer mass + mass of encapsulated DOX)] × 100.

Assuming quantitative recovery of the initial 14 mg polymer fraction, comprising 10 mg PLA-His and 4 mg PEGME-b-PDLLA, the nominal drug loading was approximately 4.99% w/w. This value was calculated from the initial formulation composition and encapsulation efficiency and was not determined independently by gravimetric analysis. For the biological experiments, the PLA-His-DOX suspension was adjusted to obtain a final DOX-equivalent concentration of 1 μg/mL. The same concentration of soluble DOX was used in the corresponding control groups. Based on the nominal drug loading of 4.99% w/w, a DOX-equivalent concentration of 1 μg/mL corresponded to approximately 20.0 μg/mL of total PLA-His-DOX nanoparticle mass. This included approximately 19.0 μg/mL of total polymer, consisting nominally of 13.6 μg/mL PLA-His and 5.4 μg/mL PEGME-b-PDLLA. These concentrations were calculated from formulation composition and encapsulation efficiency and were not measured directly in the final cell-culture suspension.

DOX release was not re-evaluated for the newly characterized batch. However, comparable PLA-His-DOX formulations prepared using the same nanoprecipitation procedure previously showed approximately 40–50% cumulative DOX release after 24 h under physiological conditions (Brzeziński et al., 2023). This previously reported release profile is provided for formulation context and should not be interpreted as a direct measurement of the batch used in the present experiments.

### 2.3 General cell culture conditions and spheroid model development

Human gastric cancer AGS cells (ATCC^®^ CRL-1739™, Manassas, USA) were cultured under standard conditions in a humidified 5% CO_2_ incubator using 25 cm^2^ tissue flasks. The medium, Ham's F-12K (Thermo Fisher Scientific, Waltham, MA, USA), was supplemented with 10% fetal bovine serum (FBS) (BioWest, Nuaillé, France), 100 U/mL penicillin, and 100 μg/mL streptomycin (BioWest, Nuaillé, France), and replaced every 2-3 days. Cells were passaged at approximately 80% confluence using 0.25% trypsin (Sigma-Aldrich, St. Louis, MO, USA). Cell morphology and conditions were monitored with an Motic AE2000 inverted light microscope (Xiamen, China) equipped with phase-contrast optics. For experiments, cells were harvested and seeded at 2 × 10^5^ cells per well in 96-well Millicell^®^ Ultra-low Attachment Plates (Merck, Darmstadt, Germany) (100 μL/well), then cultured for 72 h before stimulation.

The recombinant CagA used in this study (kindly provided by Antonello Covacci, IRIS, Siena, Italy) was the internal A17 fragment derived from *H. pylori* strain CCUG 17874 (Culture Collection University of Gothenburg, Gothenburg, Sweden). The fragment corresponded to amino acid residues Glu748–Glu977 of the CagA precursor and contained approximately 230 CagA-derived amino acids. It was expressed in *Escherichia coli* using the pEX34b expression system as a fusion protein with the N-terminal 98-amino-acid fragment of bacteriophage MS2 polymerase and was purified and stored as previously described (Covacci et al., 1993; Xiang et al., 1993). The recombinant preparation was used at a final concentration of 1 μg/mL.

*H. pylori* LPS was obtained from the reference *H. pylori* strain CCUG 17874. Whole-cell material was pretreated with proteinase K, and crude LPS was isolated using hot phenol–water extraction with 45% aqueous phenol at 68 °C for 30 min. The preparation was subsequently treated with RNase A, DNase II, and proteinase K to reduce nucleic acid and protein contamination and was purified by ultracentrifugation at 100,000 × *g* for 18 h at 4 °C, as described previously (Mattsson et al., 1998; Mnich et al., 2016; Gonciarz et al., 2019a). Previous characterization of LPS preparation obtained using this procedure demonstrated a residual protein content below 1%, as determined using Peterson's modification of the Lowry method. LPS was further analyzed by sodium dodecyl sulfate–polyacrylamide gel electrophoresis followed by silver staining (Mattsson et al., 1998). Residual nucleic acid concentration was not independently quantified after enzymatic purification. LPS has been lyophilized and stored in room temperature.

The working LPS solution (25 ng/mL) has been selected experimentally in the previous study (Gonciarz et al., 2019a, 2021). Standard *E. coli* LPS O55:B5 (Merck, Darmstadt, Germany) was used in the same concentration.

Cells were exposed to CagA or LPS for 24 or 72 h, and/or remaining components as follows: bacterial components alone (*H. pylori* CagA LPS *H. pylori*, or LPS *E. coli*), DOX (Sigma-Aldrich, Darmstadt, Germany), or PLA- His-DOX (as previously described, Jaroniek et al., 2025) at 1 μg/mL, with or without bacterial components (*H. pylori*, CagA LPS *H. pylori*, or LPS *E. coli*), and with DOX or PLA-His-DOX.

### 2.4 Visualization and morphometric analysis of spheroids

The influence of the tested conditions on spheroid morphology and three-dimensional organization was assessed using an automated inverted fluorescence microscope, the Leica Mica Microhub (Leica Microsystems, Wetzlar, Germany). Spheroids were imaged after 24 and 72 h of exposure to the tested compounds. Cells were stained with 4′, 6-diamidino-2-phenylindole (DAPI) for 20 min to visualize cell nuclei. Imaging was performed using DAPI fluorescence settings with excitation at 358 nm and emission at 461 nm, as well as transmitted light. Z-stack images composed of six optical sections were acquired and merged to visualize 3D organization of spheroids. Depending on the sample, the optical section interval ranged from 9.84 to 14.69 μm, corresponding to a total imaging depth ranging from 49.21 to 73.45 μm. Four spheroids were analyzed for each experimental condition in each of three independent experiments. DOX-associated fluorescence was assessed in the acquired Z-stack images. Because the polymeric carrier was not independently labeled, the detected fluorescence was interpreted as the distribution of soluble or nanoparticle-delivered DOX and did not allow intact NPs to be distinguished from released DOX.

Transmitted-light images were subjected to morphometric analysis using ImageJ software (National Institutes of Health, Bethesda, MD, USA; https://imagej.net/). After spatial calibration using the microscope image metadata, spheroid contours were delineated and the following parameters were quantified: spheroid diameter, total projected spheroid area, and circularity. Spheroid diameter was expressed in micrometers, whereas the projected area was expressed in square micrometers and represented the total two-dimensional area enclosed by the spheroid contour. Circularity was calculated by ImageJ according to the formula: Circularity = 4π × area/perimeter^2^.

Circularity values range from 0 to 1, with values approaching 1 indicating a more circular and regular spheroid outline. The same calibration, contour-delineation procedure, and measurement settings were applied to all experimental groups and time points.

The distribution of DOX-associated fluorescence within the spheroids was assessed after exposure to soluble DOX or PLA-His-DOX nanoparticles. Nanoparticle carrier was not independently labeled; the detected fluorescence was interpreted as the distribution of soluble or nanoparticle-delivered DOX rather than direct evidence of intact nanoparticle uptake. The DOX fluorescence signal was detected using excitation and emission wavelengths of 470 and 595 nm, respectively. Image acquisition and visualization were performed using Leica LAS X software (Leica Microsystems).

### 2.5 Cell cytotoxicity assay

The influence of experimental conditions on cell cytotoxicity was assessed using the MTT [(3-(4,5-dimethylthiazol-2-yl) 2,5-diphenyltetrazolium bromide)] assay based on the cells' capacity to reduce MTT to formazan crystals, in accordance with guidelines set by the Food and Drug Administration and the International Organization for Standardization (ISO) standard 10993-5:2009 Biological evaluation of medical devices Part 5: Tests for *in vitro* cytotoxicity as previously described (Brzeziński et al., 2023). The cell cytotoxicity was shown as percentage of viable cells.

### 2.6 Preparation of cells for immunohistochemical staining

The spheroid cultures were prepared and stimulated as described in Section 2.3. Subsequently, the spheroids were transferred to sterile black 96-well plates with glass bottoms (Corning, NY, USA). The culture medium was removed, and cells were treated with standard trypsin solution to dissociate the spheroids. Then, cells were washed twice with phosphate-buffered saline (PBS) (BioWest, Nuaillé, France), and cells were fixed with 4% paraformaldehyde for 15 min at room temperature. A 0.02% solution of Triton X-100 (Merck, Darmstadt, Germany) was used for cell permeabilization for 10 min at room temperature. Non-specific binding sites were blocked by incubating the samples with 3% bovine serum albumin (BSA) (Merck, Darmstadt, Germany) in PBS for 30 min to 1 h at room temperature.

### 2.7 Apoptosis

To determine the level of apoptosis, the deoxynucleotidyl transferase dUTP nick end labeling (TUNEL) assay was used (Thermo Fisher Scientific, Waltham, MA, USA). Cells were prepared as described in Section 2.3 and subjected to DNA damage and cleaved caspase-9 testing. To determine DNA damage, the cell cultures were incubated with a primary antibody against phosphorylated H2AX (Ser139) (Thermo Fisher Scientific, Waltham, MA, USA) and then stained with secondary antibodies fluorescently labeled with Alexa Fluor 488 (Thermo Fisher Scientific, Waltham, MA, USA). The presence of cleaved caspase 9 was determined using primary anti-cleaved caspase 9 antibodies (Thermo Fisher Scientific, Waltham, MA, USA) and then secondary Alexa Fluor 633 labeled antibodies (Thermo Fisher Scientific, Waltham, MA, USA). The fluorescence intensity was measured using a multifunctional spectrophotometer, SpectraMax i3 (Molecular Devices, San Jose, CA, USA). The measurements were performed using the specified excitation and emission parameters: excitation-782 nm and emission- 805 nm for TUNEL assay, excitation-495 nm and emission-519 nm for DNA damage, and excitation- 631 nm and emission-650 nm for cleaved caspase 9 detection. Data obtained under the tested conditions were calculated relative to the medium and presented as relative fluorescence units (RFU). Three independent experiments were carried out in triplicate.

### 2.8 Cell proliferation

Cell proliferation was assessed using the CyQUANT Cell Proliferation Assay Kit (Invitrogen, Carlsbad, CA, USA) based on the fluorescence probe according to the manufacturer's instructions. Fluorescence was measured with the multifunctional spectrophotometer SpectraMax i3 (Molecular Devices, San Jose, CA, USA) at excitation and emission wavelengths: 782 nm and 805 nm, respectively. Each experimental condition was tested in triplicate. Proliferation intensity was shown as relative fluorescence units (RFU) relative to the medium.

### 2.9 Fluorescence-based assessment of cell membrane bound and peroxidation markers

Spheroids were prepared and stimulated as described in Section 2.3 while for staining as in Section 2.6. Cells were stained using antibodies against zonula occludens ZO-1 fluorescently labeled with AlexaFluor 546 (excitation 561 nm, emission 572 nm) (Santa Cruz Biotechnology, Dallas, TX, USA) or with anti-occludin antibodies labeled with AlexaFluor 790 (excitation 782 nm, emission 805 nm) (Santa Cruz Biotechnology, Dallas, TX, USA). Lipid peroxidation was detected based on hydroxynonenal (4HNE) staining using primary anti-4HNE antibodies (Thermo Fisher Scientific, Waltham, MA, USA) and then fluorescently labeled secondary antibodies AlexaFluor 568 (excitation 579 nm, emission 603 nm) (Thermo Fisher Scientific, Waltham, MA, USA). The fluorescence intensity was measured using a multifunctional spectrophotometer, SpectraMax i3 (Molecular Devices, San Jose, CA, USA), at the appropriate emission and excitation wavelengths for each fluorochrome. Data obtained under the tested conditions were calculated relative to the medium and presented as relative fluorescence units (RFU). Three independent experiments were carried out in triplicate.

### 2.10 Cytokine production

Cell culture supernatants from spheroid cultures exposed to the experimental conditions described in Section 2.3 were collected and used to determine IL-1β and IL-8. The concentration of each cytokine was assessed using commercially available Enzyme-Linked Immunosorbent Assay (ELISA) kit according to the manufacturer's procedure (Thermo Fisher Scientific, Waltham, MA, USA). The analytical sensitivity was 15.6 pg/mL for IL-1β and 5 pg/mL for IL-8.

### 2.11 Short-term biocompatibility assessment of blank PLA-His NPs in *Cavia porcellus*

Ethical Statements: All animal experiments adhered to the EU directive (Directive 2010/63/EU of the European Parliament and Council, dated 22 September 2010) for animal protection in scientific research. These studies received approval from the Local Ethics Committee (LKE9) for Animal Experiments at the Medical University of Lodz, Poland. They were authorized by the Ministry of Science and Higher Education (ŁB 16/234/2022). Both male and female, three-month-old, pathogen-free *Cavia porcellus* (guinea pigs) (5 animals/group) were bred and housed in the Animal House at the Faculty of Biology and Environmental Protection, University of Lodz. They were kept in air-conditioned rooms at 20–24°C with cages offering continuous access to water and food pellets, on a 12-h light/dark cycle. 3 month-old, pathogen-free male and female *Cavia porcellus* guinea pigs were bred and maintained in the Animal House of the Faculty of Biology and Environmental Protection, University of Lodz, Poland. The animals were housed in air-conditioned rooms at 20–24 °C under a 12-h light/12-h dark cycle, with ad libitum access to drinking water and standard food pellets.

The number of animals was determined following the 3R principle and was estimated in advance using G^*^Power software. Each group contained five animals, as calculated in G^*^Power with a type I error probability of α = 0.05 and a statistical power of 1 – β = 0.80. Animals were allocated to the experimental groups using sex-stratified randomization. Male and female guinea pigs were first separated into sex-specific strata and then assigned to the control or PLA-His nanoparticle group using a computer-generated random number sequence. The sex distribution was balanced between the groups: one group included three males and two females, while the other included two males and three females. Before administration, the animals underwent clinical examination and body weight measurement. Guinea pigs in the experimental groups received orally 1 mL of blank PLA-His NPs suspension at a concentration of 1 μg/mL. The administered dose was therefore 1 μg per animal. Based on the body weight range of 400–600 g previously reported for age-matched guinea pigs maintained in the same animal facility, the administered dose was estimated to correspond to approximately 1.67–2.50 μg/kg body weight, with a nominal dose of approximately 2.0 μg/kg for an animal weighing 500 g. NPs did not contain DOX. Control animals received orally 1 mL of sterile 0.85% sodium chloride solution. The NPs suspension used for biological testing was prepared in accordance with PN-EN ISO 10993-12:2012. Animals were monitored from administration to euthanasia based on general condition, physical activity, behavioral symptoms, food and water intake, skin and fur condition, body temperature, heart rate, respiration, diarrhea, and other visible adverse reactions. Body weight was recorded before administration and at the experimental endpoint.

Animals were euthanized 24 or 72 h after oral administration using an overdose of sodium barbiturate (Morbital, Biowet, Puławy, Poland). Blood was collected at the experimental endpoint, and serum was isolated for analyses of biochemical, inflammatory, renal, and immune responses. Tissue samples from the liver, kidneys, and spleen were collected for histological examination. Gastric and intestinal tissues were not collected.

Serum concentrations of C-reactive protein (CRP), (TNF-α), (IL-1β), total immunoglobulin E (IgE), and cystatin C were determined using commercially available enzyme-linked immunosorbent assay kits according to the manufacturers' instructions as previously described (Gonciarz et al., 2024). The analytical sensitivities were 0.15 ng/mL for CRP (Merck, St. Louis, MO, USA), 15.6 pg/mL for TNF-α, 8 pg/mL for IL-1β, 4 ng/mL for IgE, and 10 pg/mL for cystatin C (Thermo Fisher Scientific, Waltham, MA, USA). Aspartate aminotransferase (AST) and alanine aminotransferase (ALT) activities were determined in serum and liver homogenates using commercially available colorimetric assays according to the manufacturer's instructions (Merck, Darmstadt, Germany).

Liver, kidney, and spleen samples were fixed, embedded in paraffin, sectioned, stained with hematoxylin and eosin, and then examined for inflammatory-cell infiltration, vascular changes, necrosis, and other histopathological abnormalities.

### 2.12 Statistical analysis

Data are presented as mean ± standard error of the mean (SEM) from three independent experiments. For each experimental condition, four technical replicates were performed within each independent experiment. The four replicate measurements were averaged to obtain one value for each independent experiment; therefore, the independent experiment was treated as the statistical unit (*n* = 3 per experimental condition). Comparisons between two groups were performed using the Mann–Whitney U test. Comparisons involving more than two groups were performed using the Kruskal–Wallis test followed by Dunn's *post hoc* test. Differences were considered statistically significant at *p* < 0.05. All statistical analyses were performed using GraphPad Prism 10 (GraphPad Software, San Diego, CA, USA).

## 3 Results

### 3.1 The influence of bacterial components and/or DOX and PLA-His-DOX on spheroid architecture, spheroidal cell viability and pro-inflammatory activity

**Figure 1:**
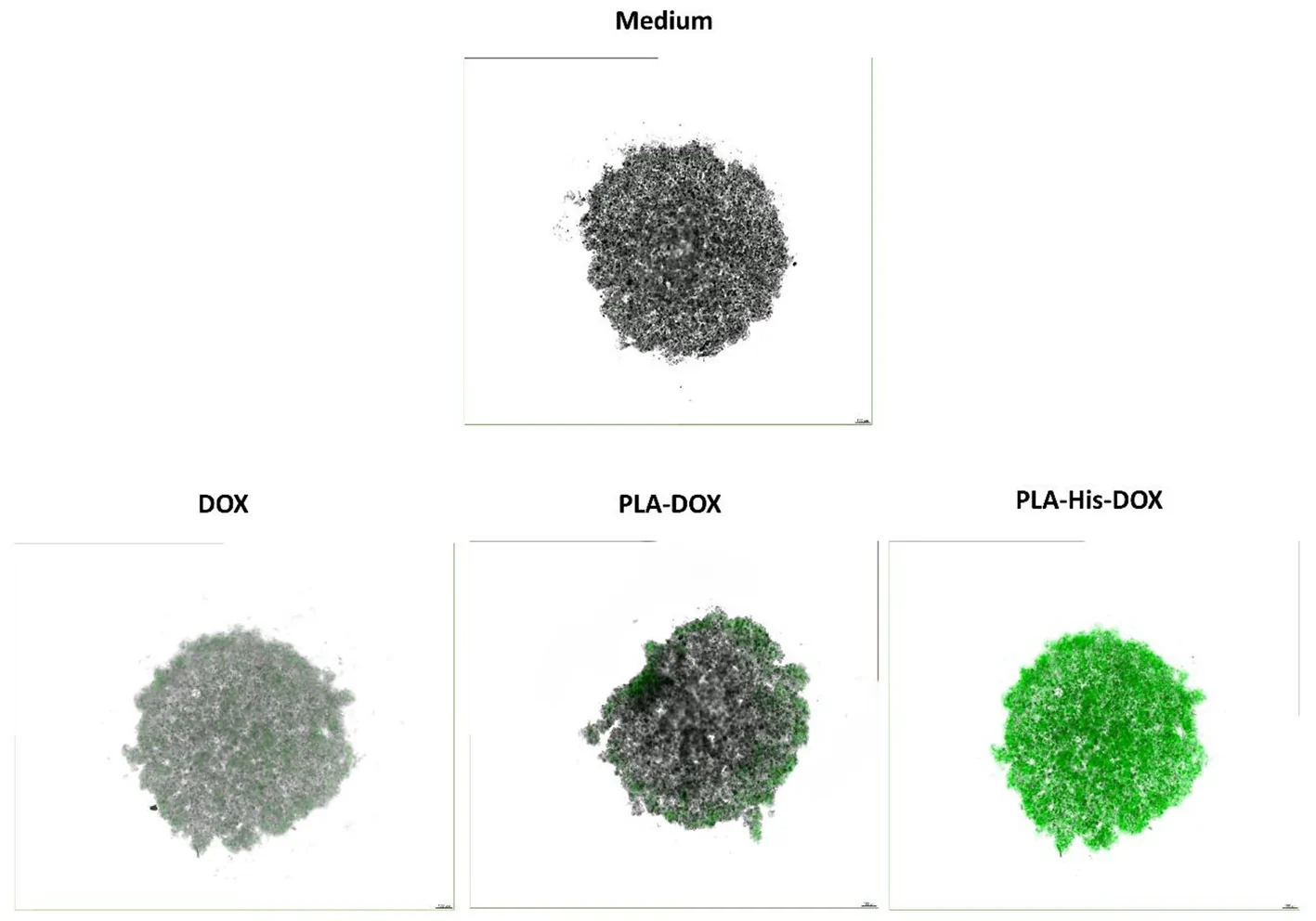
Cellular penetration of soluble and encapsulated DOX. The penetration of soluble DOX or DOX from tested nanoparticles was visualized using the Leica Mica Microhub microscope (Leica Microsystems, Wetzlar, Germany) at wavelength parameters corresponding to the fluorescence of DOX. DOX, doxorubicin; PLA-DOX, NPs PLA-OH loaded with DOX; PLA-His-DOX, NPs PLA modified with histamine (His) and loaded with DOX. Data are presented as mean ± SEM from three independent experiments (*n* = 3). Four technical replicates were analyzed for each condition in each experiment and averaged before statistical analysis.

AGS cells developed characteristic 3D spheroid architecture *in vitro* with exposed core, quiescent zone and rim with cells capable of spreading *in vitro* as shown in Figure 1. The intracellular penetration of soluble DOX or DOX released from PLA-DOX or PLA-His-DOX into the AGS-derived spheroids has been shown. The penetration effect of DOX was highest in the case of DOX released from PLA-His-DOX NPs compared to soluble DOX or PLA-DOX (Figure 1). Representative Z-stack images showed DOX-associated fluorescence within AGS spheroids exposed to soluble DOX, PLA-DOX, or PLA-His-DOX. The fluorescence signal detected after PLA-His-DOX treatment extended from the peripheral layers toward the inner spheroid region and appeared more intense than that observed after exposure to soluble DOX or PLA-DOX. These observations indicate enhanced trans spheroidal distribution of DOX delivered by PLA-His-DOX under the tested conditions (Figure 1).

**Figure 2:**
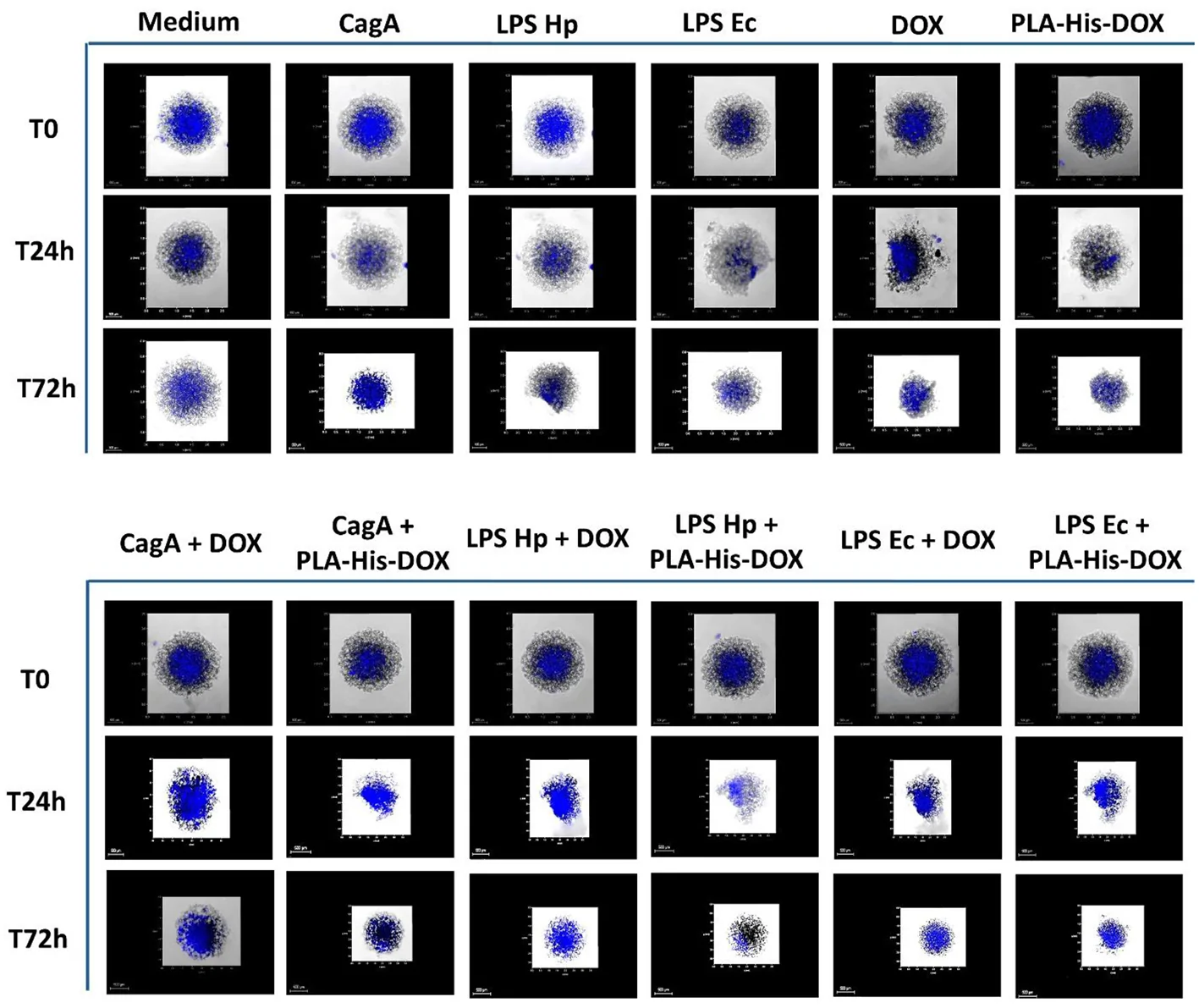
The influence of bacterial components and DOX or PLA-His-DOX on the architecture of developed spheroids. The images were obtained using the Leica Mica Microhub microscope (Leica Microsystems, Wetzlar, Germany). The median values were assessed using the Kruskal-Wallis test, followed by Dunn's *post-hoc* test with a significance level of *p* < 0.05. * cells in medium vs. cells exposed to bacterial components, DOX, PLA-His-DOX alone or in co-cultures, ■ unstimulated cells at T0 vs. cells exposed to bacterial components and/or DOX or PLA-His-DOX. CagA, cytotoxin-associated gene A protein; LPS Hp, *H. pylori* lipopolysaccharide; LPS Ec*, E. coli* lipopolisaccharide; DOX, doxorubicin; PLA-His-DOX, NPs PLA modified with histamine (His) and loaded with DOX. Data are presented as mean ± SEM from three independent experiments (*n* = 3). Four technical replicates were analyzed for each condition in each experiment and averaged before statistical analysis.

**Figure 3:**
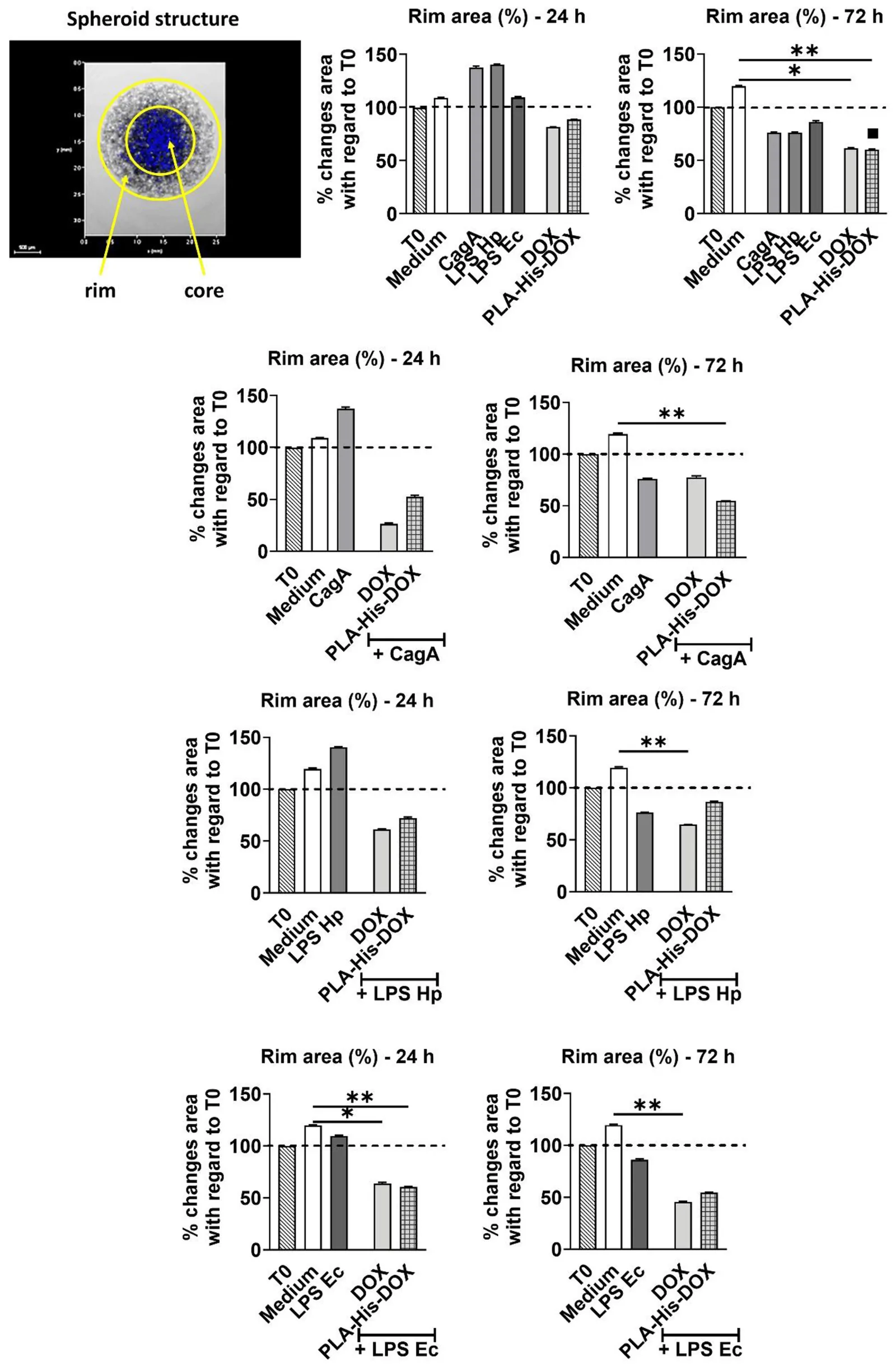
The influence of bacterial components and DOX or PLA-His-DOX on the architecture of developed spheroids. Changes in rim area after treatment with bacterial components, DOX, or PLA-His-DOX. The median values were assessed using the Kruskal-Wallis test, followed by Dunn's *post-hoc* test with a significance level of *p* < 0.05. * cells in medium vs. cells exposed to bacterial components, DOX, PLA-His-DOX alone or in co-cultures, ■ unstimulated cells at T0 vs. cells exposed to bacterial components and/or DOX or PLA-His-DOX. CagA, cytotoxin-associated gene A protein; LPS Hp, *H. pylori* lipopolysaccharide; LPS Ec*, E. coli* lipopolisaccharide; DOX, doxorubicin; PLA-His-DOX, NPs PLA modified with histamine (His) and loaded with DOX. Data are presented as mean ± SEM from three independent experiments (*n* = 3). Four technical replicates were analyzed for each condition in each experiment and averaged before statistical analysis. ^*^p < 0.05, ^**^p < 0.01, ^***^p < 0.001.

The architecture of spheroids was influenced by bacterial components particularly *H. pylori* CagA or LPS *H. pylori*, and less by LPS *E. coli* after 24 and 72 h of cell exposure. In 24 h cultures the spheroids showed tendency to increased rim area after cell exposure to CagA and LPS *H. pylori*. There was no such effect in cell cultures treated with soluble DOX or PLA-His-DOX. After 72 h the rim area was diminished in all tested culture variants compared to control cell culture in medium alone (Figures 2, 3). Furthermore, in the presence of soluble DOX or particularly PLA-His-DOX the broader rim zone in spheroids developed in CagA or *H. pylori* LPS conditions was diminished (Figures 2, 3). In both assessment points after 24h or 72h an increased amount of ZO-1 tight junction protein has been shown in spheroid cultures with DOX or PLA-His-DOX, while occludin remained at unchanged level (Supplementary Figure 5).

Morphometric analysis demonstrated that the total projected area was more sensitive to the experimental conditions than spheroid diameter or circularity (Supplementary Figures 2–4). After 24 h, spheroids maintained in medium had a mean projected area of approximately 3.4 × 106 μm^2^. Exposure to *H. pylori* CagA, *H. pylori* LPS, or *E. coli* LPS alone caused only moderate changes in this parameter. In contrast, the addition of DOX or PLA-His-DOX in the presence of bacterial components reduced the projected spheroid area. Significant reductions were observed in selected comparisons involving *H. pylori* CagA, *H. pylori* LPS or *E. coli* LPS, as indicated in Supplementary Figure 2.

After 72 h, the projected area of untreated spheroids remained approximately 3.4–3.5 × 106 μm^2^, whereas markedly lower values were showed in most spheroid groups exposed to bacterial components, DOX, PLA-His-DOX, or their combinations. *H. pylori* CagA and *H. pylori* LPS alone reduced the projected area to approximately 1.6–1.7 × 106 μm^2^. In the presence of *H. pylori* CagA or *H. pylori* LPS, spheroids treated with DOX or PLA-His-DOX showed projected areas of approximately 2.2–2.6 × 106 μm^2^. Exposure of spheroids to *E. coli* LPS, either alone or combined with DOX or PLA-His-DOX, resulted in projected areas of approximately 1.5–1.8 × 106 μm^2^. Statistically significant differences between tested variants *vs*. control (cell culture in medium alone) are shown in Supplementary Figure 2.

The mean diameter of untreated spheroids was approximately 1,030–1,050 μm after both 24 and 72 h (Supplementary Figure 3). Exposure of spheroids to bacterial components alone caused minor changes in spheroid diameter. DOX and PLA-His-DOX administered without bacterial components slightly reduced sthe spheroid diameter to approximately 950–980 μm. In contrast, the combination of DOX or PLA-His-DOX with *H. pylori* CagA or *H. pylori* LPS showed tendency to increase spheroid diameter, reaching approximately 1.150–1.270 μm. This tendency was not observed in spheroids exposed to *E. coli* LPS, for which the diameter remained close to or below to control. None of the differences in spheroid diameter reached statistical significance.

Circularity values ranged from 0.13 to 0.35, indicating that spheroids did not exhibit a perfect circular outline (Supplementary Figure 4). Untreated spheroids showed circularity values of approximately 0.24–0.26. At 24 h, *H. pylori* CagA reduced spheroid circularity to approximately 0.15, whereas *E. coli* LPS increased spheroid circularity to approximately 0.35. In the presence of DOX or PLA-His-DOX and *H. pylori* CagA or *H. pylori* LPS spheroid circularity was lowered ranging from approximately 0.13 to 0.18. After 72 h, a significant reduction in circularity was observed in the *H. pylori* CagA-conditioned groups treated with DOX or PLA-His-DOX, as indicated in Supplementary Figure 4. No other consistent statistically significant changes in spheroid circularity were detected.

Spheroidal cells did not show increased proliferating activity in any culture variant (Supplementary Figure 5).

In parallel metabolic activity of AGS cells was assessed using MTT reduction assay after cell exposure to bacterial components (CagA *H. pylori*, LPS *H. pylori*, LPS *E. coli*) and/or DOX or PLA-His-DOX. After 24 h only metabolic activity of spheroid cells exposed to PLA-His-DOX was diminished (below 70% of viable cells), while after 72 h in all cell culture variants reduction of MTT and therefore cell viability was diminished compared to control cell culture in medium alone.

**Figure 4:**
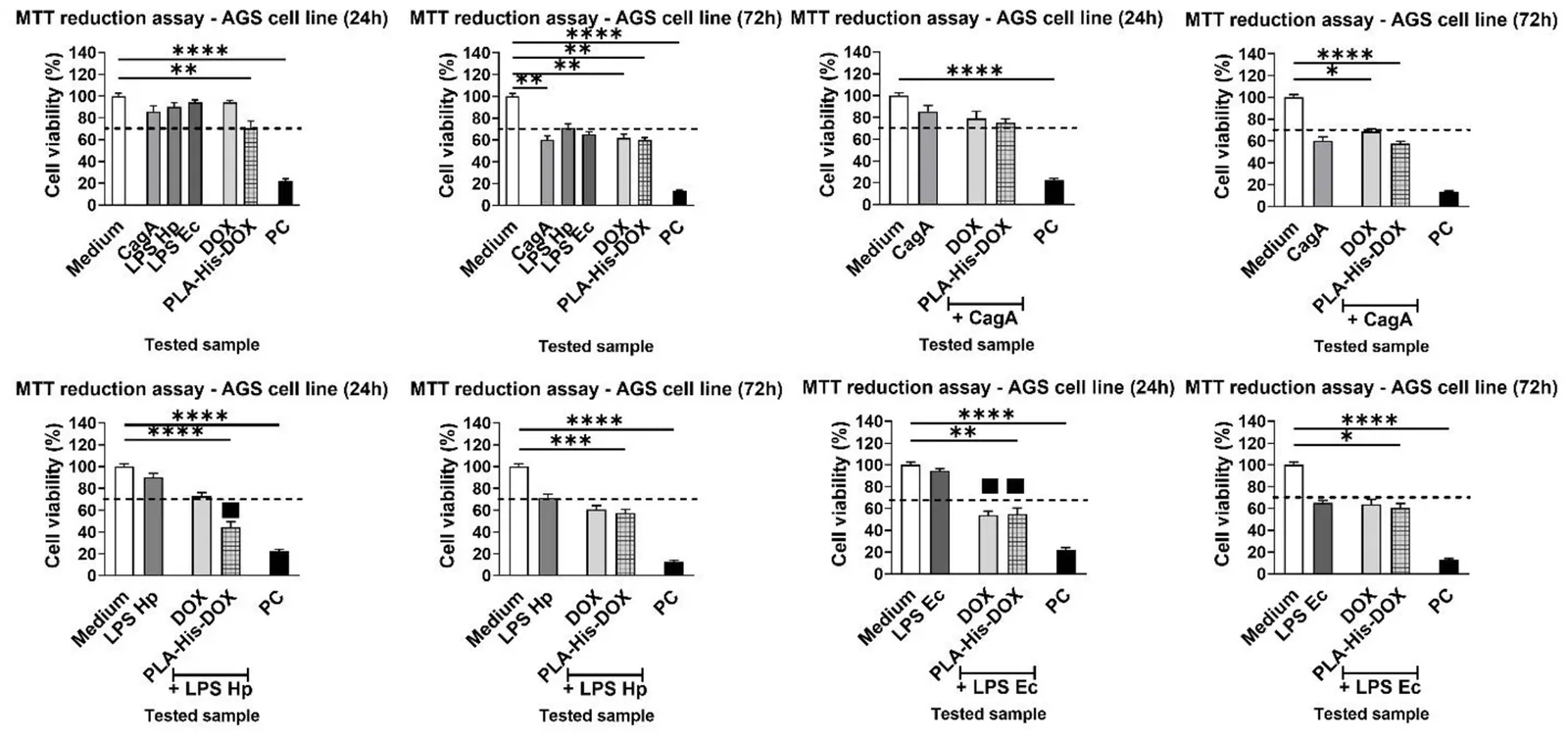
The influence of bacterial components and DOX or PLA-His-DOX on spheroid cell viability. The median values were assessed using the U Mann-Whitney test or Kruskal-Wallis test, followed by Dunn's *post-hoc* test with a significance level of *p* < 0.05. * cells in medium vs. cells exposed to bacterial components, DOX, PLA-His-DOX alone or in co-cultures ■ cells exposed to bacterial component vs. cells exposed to bacterial component and DOX or PLA-His-DOX. CagA, cytotoxin-associated gene A protein; LPS Hp, *H. pylori* lipopolisaccharide; LPS Ec, *E. coli* lipopolisaccharide DOX, doxorubicin; PLA-His-DOX, NPs PLA modified with histamine (His) and loaded with DOX; Positive control contained 2 μg/mL soluble DOX and was used to confirm assay responsiveness. Data are presented as mean ± SEM from three independent experiments (*n* = 3). Four technical replicates were analyzed for each condition in each experiment and averaged before statistical analysis. ^*^p < 0.05, ^**^p < 0.01, ^***^p < 0.001, ^***^p < 0.0001.

In spheroid co-cultures, treated for 24 h or 72h with CagA, *H. pylori* LPS or *E. coli* LPS and DOX or PLA-His-DOX the cell metabolic activity was further diminished (Figure 4).

**Figure 5:**
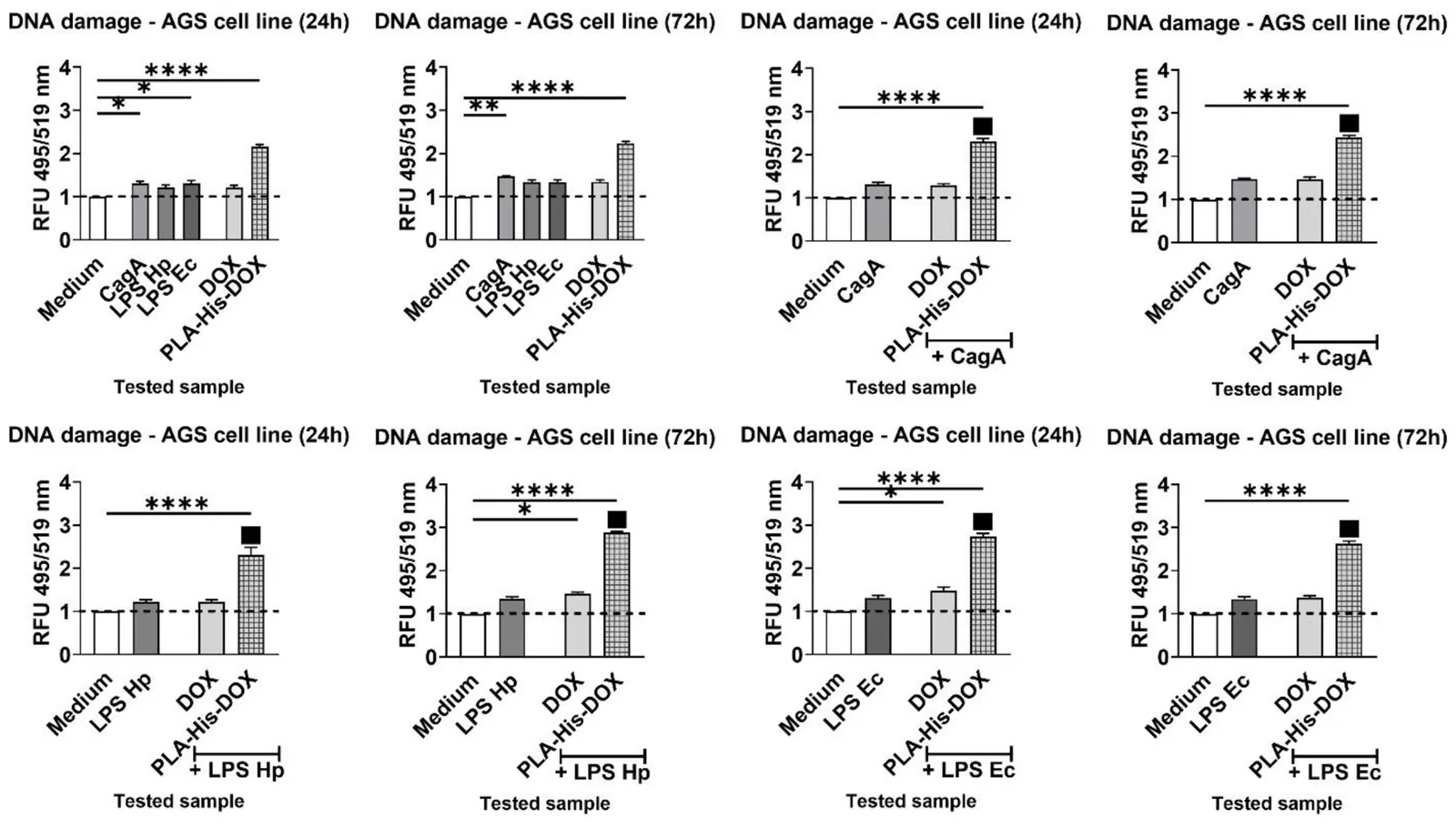
DNA damage in spheroid cultures. Spheroids were exposed to bacterial components, DOX or PLA-His-DOX alone or in co-cultures. The median values were assessed using the U Mann-Whitney test or Kruskal-Wallis test, followed by Dunn's *post-hoc* test with a significance level of *p* < 0.05. * cells in medium vs. cells exposed to bacterial components, DOX or PLA-His-DOX, ■ cells exposed to bacterial vs. cells exposed to bacterial component and DOX or PLA-His-DOX. CagA, cytotoxin-associated gene A; LPS Hp, *H. pylori* lipopolisaccharide; LPS Ec, *E. coli* lipopolisaccharide DOX, doxorubicin; PLA-His-DOX, NPs PLA modified with histamine (His) and loaded with DOX. Data are presented as mean ± SEM from three independent experiments (*n* = 3). Four technical replicates were analyzed for each condition in each experiment and averaged before statistical analysis. ^*^p < 0.05, ^**^p < 0.01, ^***^p < 0.001, ^***^p < 0.0001.

**Figure 6:**
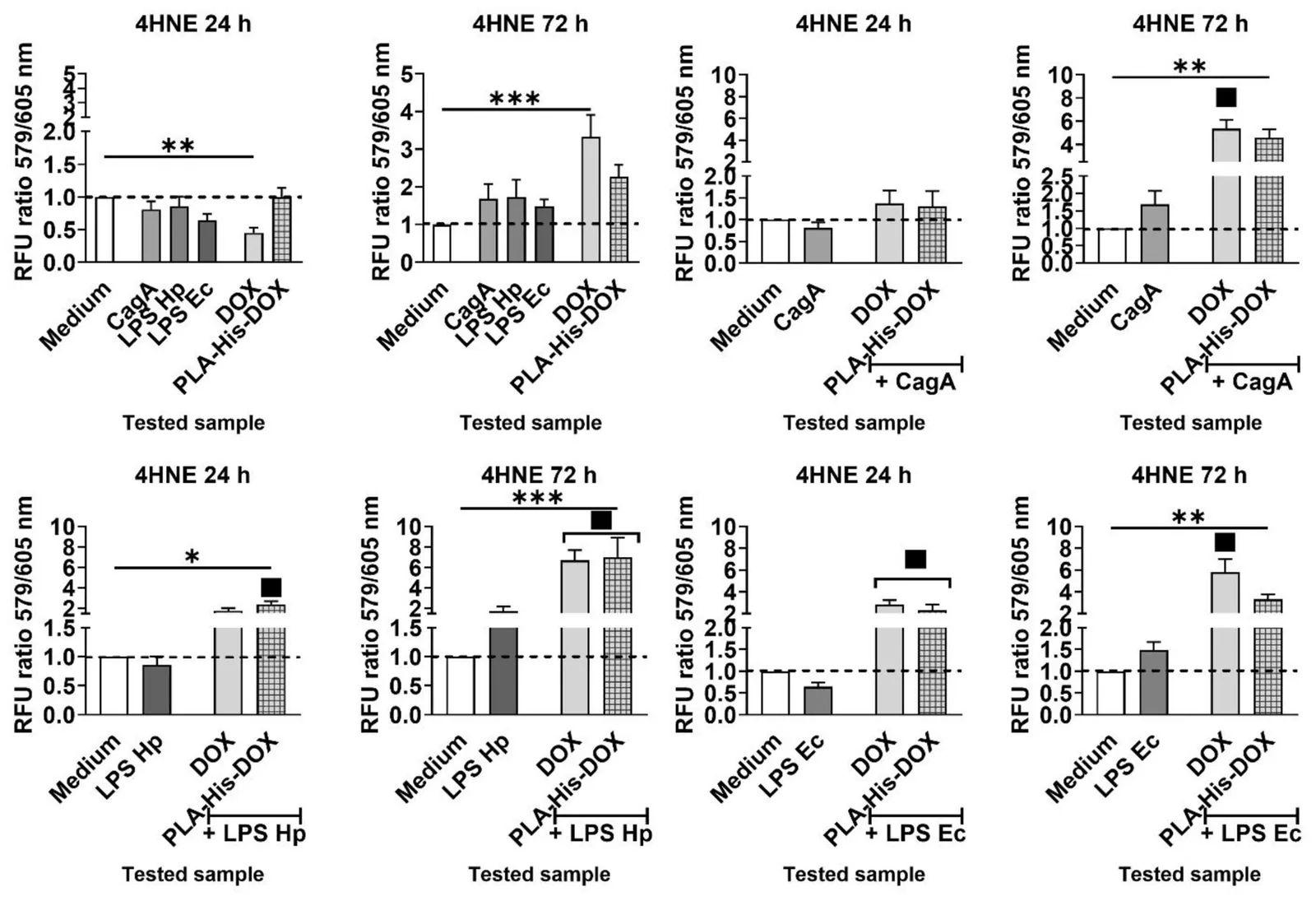
Oxidative stress markers in spheroid cultures. Spheroids were exposed to bacterial components, DOX or PLA-His-DOX alone or in co-cultures. The median values were assessed using the U Mann-Whitney test or Kruskal-Wallis test, followed by Dunn's *post-hoc* test with a significance level of *p* < 0.05. * cells in medium vs. cells exposed to bacterial components, DOX or PLA-His-DOX, ■ cells exposed to bacterial vs. cells exposed to bacterial component and DOX or PLA-His-DOX. CagA, cytotoxin-associated gene A; LPS Hp, *H. pylori* lipopolisaccharide; LPS Ec, *E. coli* lipopolisaccharide DOX, doxorubicin; PLA-His-DOX, NPs PLA modified with histamine (His) and loaded with DOX. Data are presented as mean ± SEM from three independent experiments (*n* = 3). Four technical replicates were analyzed for each condition in each experiment and averaged before statistical analysis. ^*^p < 0.05, ^**^p < 0.01, ^***^p < 0.001, ^***^p < 0.0001.

Increased DNA damage was shown in spheroid cultures exposed for 24h or 72h to bacterial components (in the case of CagA, the difference was significant), DOX, PLA-His-DOX or in co-cultures of spheroids with bacterial components and PLA-His-DOX or DOX (Figure 5). The observed DNA damage was related to enhanced oxidative stress as shown by elevated 4HNE (Figure 6). PLA-His-DOX alone or in combination with bacterial components showed the strongest effect.

**Figure 7:**
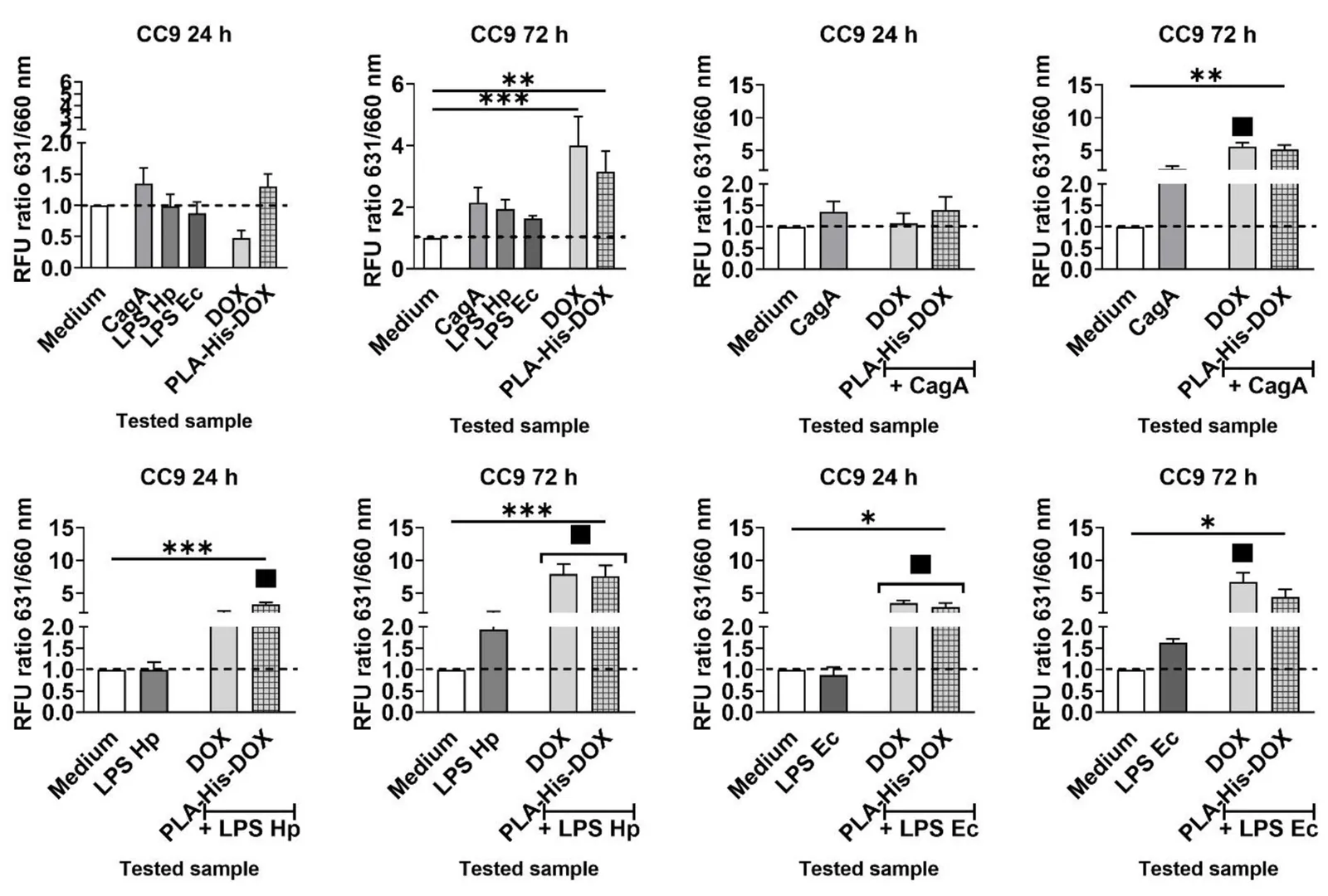
Evaluation of cleaved caspase-9 (CC9) on spheroid cultures exposed to bacterial components and/or DOX or PLA-His-DOX. The median values were assessed using the U Mann-Whitney test or Kruskal-Wallis test, followed by Dunn's *post-hoc* test with a significance level of *p* < 0.05. * cells in medium vs. cells exposed to bacterial components and/or DOX or PLA-His-DOX, ■ cells exposed to bacterial component vs. cells exposed to bacterial component and DOX or PLA-His-DOX. CagA, cytotoxin-associated gene A protein; LPS Hp, *H. pylori* lipopolisaccharide; LPS Ec, *E. coli* lipopolisaccharide DOX, doxorubicin; PLA-His-DOX, NPs PLA modified with histamine (His) and loaded with DOX. Data are presented as mean ± SEM from three independent experiments (*n* = 3). Four technical replicates were analyzed for each condition in each experiment and averaged before statistical analysis. ^*^p < 0.05, ^**^p < 0.01, ^***^p < 0.001, ^***^p < 0.0001.

**Figure 8:**
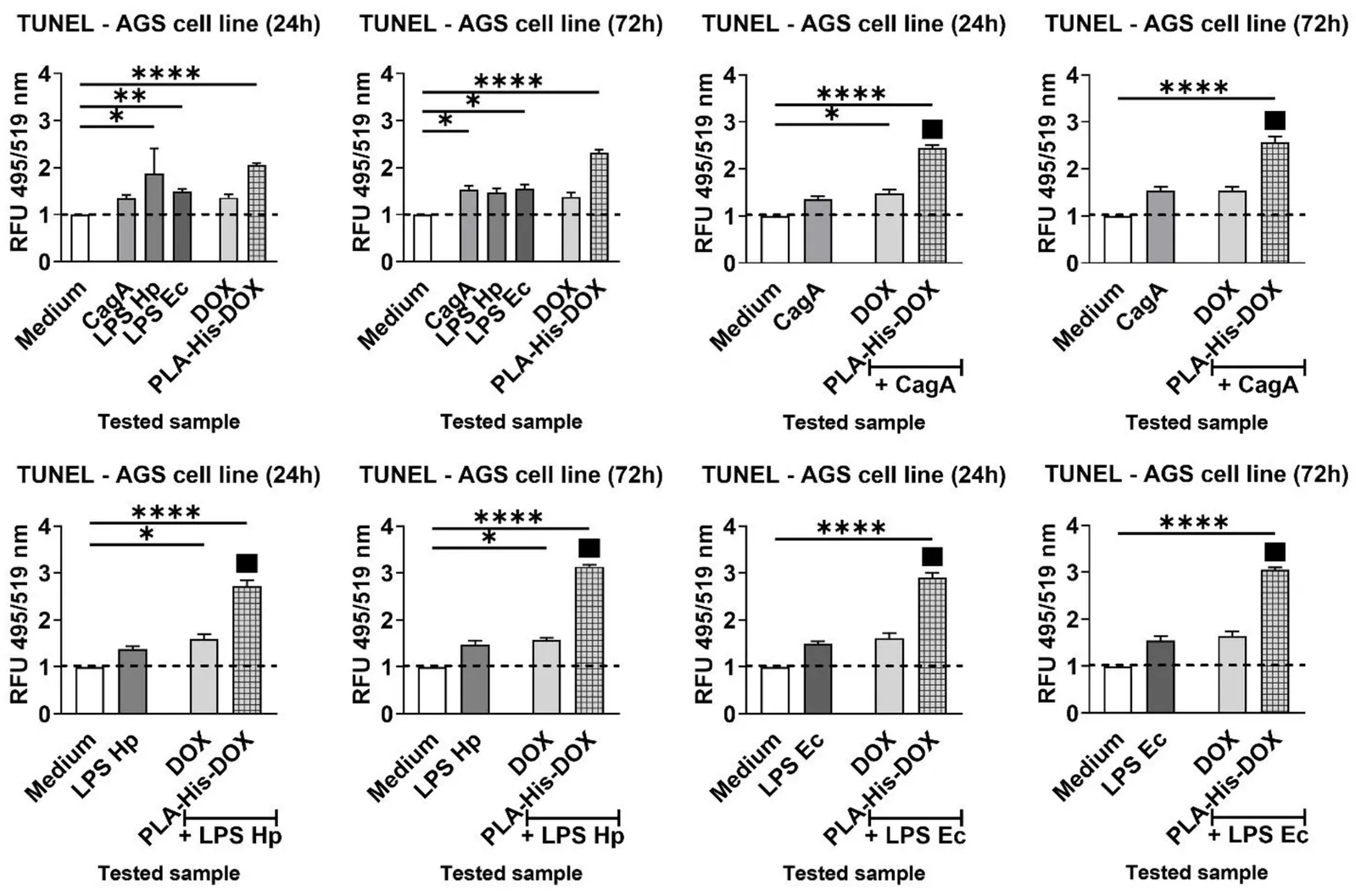
Evaluation of apoptosis on spheroid cultures exposed to bacterial components and/or DOX or PLA-His-DOX. The median values were assessed using the U Mann-Whitney test or Kruskal-Wallis test, followed by Dunn's *post-hoc* test with a significance level of *p* < 0.05. * cells in medium vs. cells exposed to bacterial components and/or DOX or PLA-His-DOX, ■ cells exposed to bacterial component vs. cells exposed to bacterial component and DOX or PLA-His-DOX. CagA, cytotoxin-associated gene A protein; LPS Hp, *H. pylori* lipopolisaccharide; LPS Ec, *E. coli* lipopolisaccharide DOX, doxorubicin; PLA-His-DOX, NPs PLA modified with histamine (His) and loaded with DOX. Data are presented as mean ± SEM from three independent experiments (*n* = 3). Four technical replicates were analyzed for each condition in each experiment and averaged before statistical analysis.

In spheroid cultures developed in the presence of bacterial components, DOX or PLA-His-DOX alone or in co-cultures: bacterial components/DOX or bacterial components/PLA-His-DOX there was a time dependent increase of cell apoptosis as shown by elevated production of cleaved caspase-9 and TUNEL assay after 72h incubation (Figures 7, 8). The strongest apoptosis signs in TUNEL assay were found in spheroid cultures containing PLA-His-DOX (Figure 8).

This study revealed no statistically significant changes in the concentrations of IL-1β or IL-8 in cell culture supernatants after exposure of spheroidal cells to bacterial components (*H. pylori* CagA, *H. pylori* LPS or *E. coli* LPS), DOX, or PLA-His-DOX for 24 or 72 h compared to cells maintained in medium alone (Supplementary Figures 6, 7).

In selected direct comparisons, lower IL-1β concentrations were detected in supernatants from spheroid cultures exposed to bacterial component combined with DOX or PLA-His-DOX than in supernatants from spheroid cultures exposed to the corresponding bacterial component alone (*p* < 0.05 with CagA vs CagA/DOX and CagA vs CagA/PLA-His-DOX; *p* < 0.05 *H. pylori* LPS vs *H. pylori* LPS/DOX; and *p* < 0.05 for *E. coli* LPS vs *E. coli* LPS/PLA-His-DOX. Lower IL-8 concentrations were detected in cell cultures containing) CagA vs CagA/PLA-His-DOX and *H. pylori* LPS or *E. coli* LPS vs corresponding LPS/DOX group (*p* < 0.05; Supplementary Figures 6, 7).

### 3.2 Systemic and local biocompatibility markers in *Cavia porcellus* inoculated *per os* with the studied PLA-His NPs

**Figure 9:**
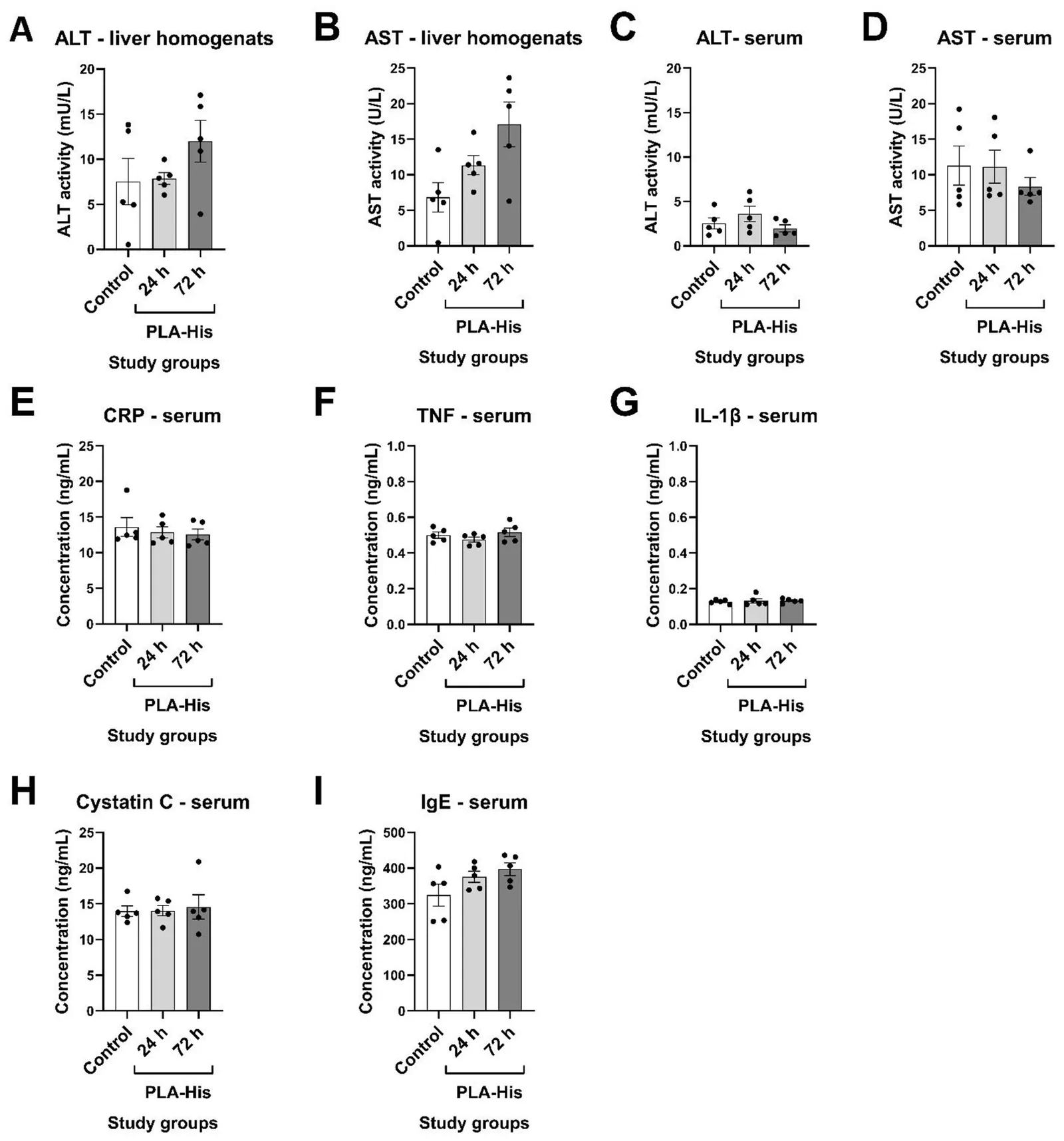
Short-term biochemical assessments in guinea pigs inoculated per os with blank PLA-His NPs. Animals receiving *per o*s tested PLA nanoparticles (NPs) with histamine (His) PLA-His or 0.85% NaCl (control animals) were terminated for isolation of tissue samples (liver) or blood collection. **(A)** The level of alanine aminotransferase (ALT) **(B)** and aspartate aminotransferase (AST) in liver tissue homogenates or serum **(C, D)**; the level of C-reactive protein CRP **(E)**, tumor necrosis factor alpha (TNF-α) **(F)** or interleukin (IL)-1ß **(G)**, cystatin C **(H)** and IgE level **(I)** in serum (ELISA). Five animals were in the group. The results are shown as median ± range.

**Figure 10:**
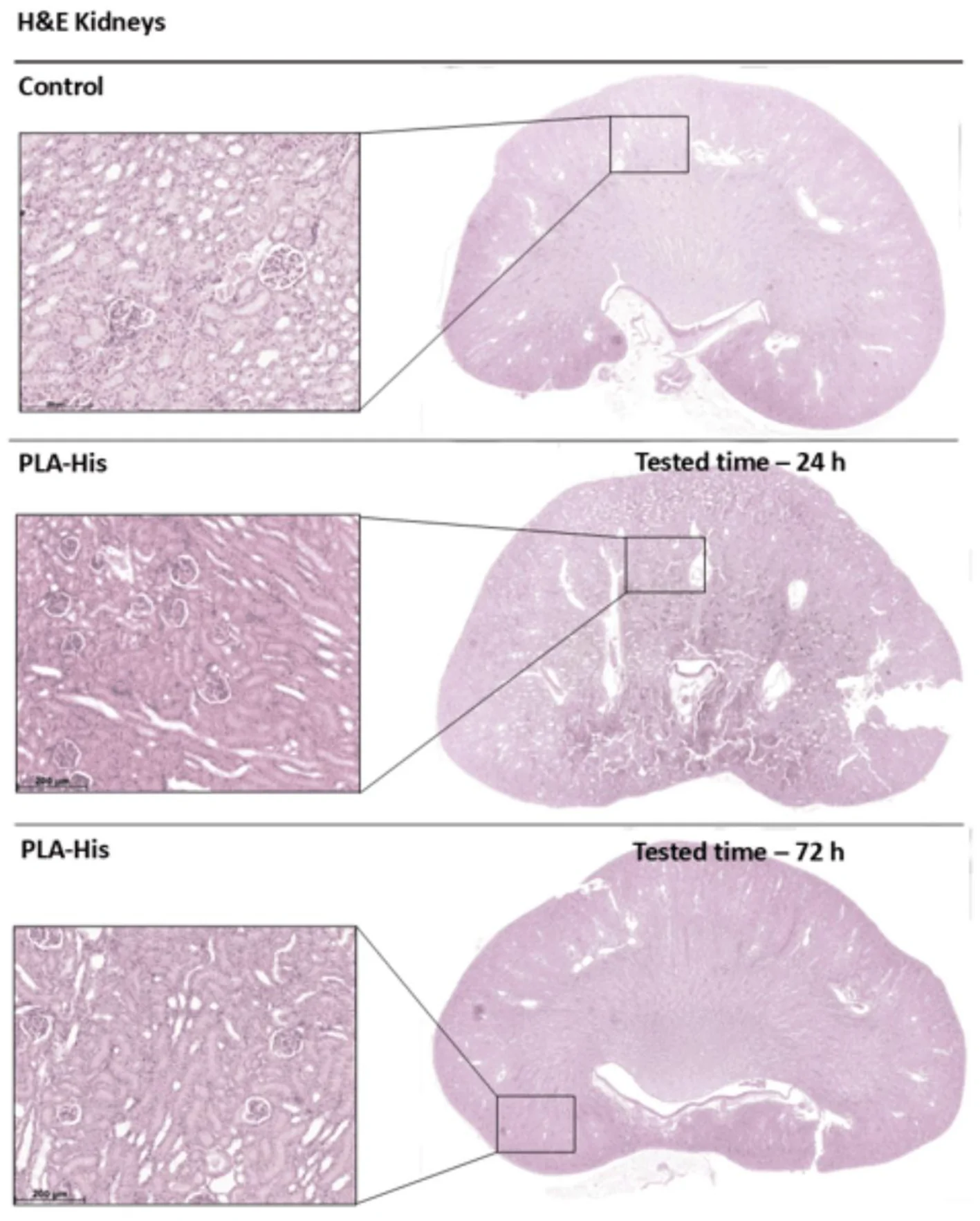
Hematoxylin-eosin (H&E) staining of kidney sections. Animals receiving *per os* tested nanoparticles (NPs) with histamine (His) PLA-His or 0.85% NaCl (control animals). Images were acquired using a Leica Mica Microhub microscope (Leica Microsystems, Wetzlar, Germany) at 10x and 40x magnification (water immersion)

**Figure 11:**
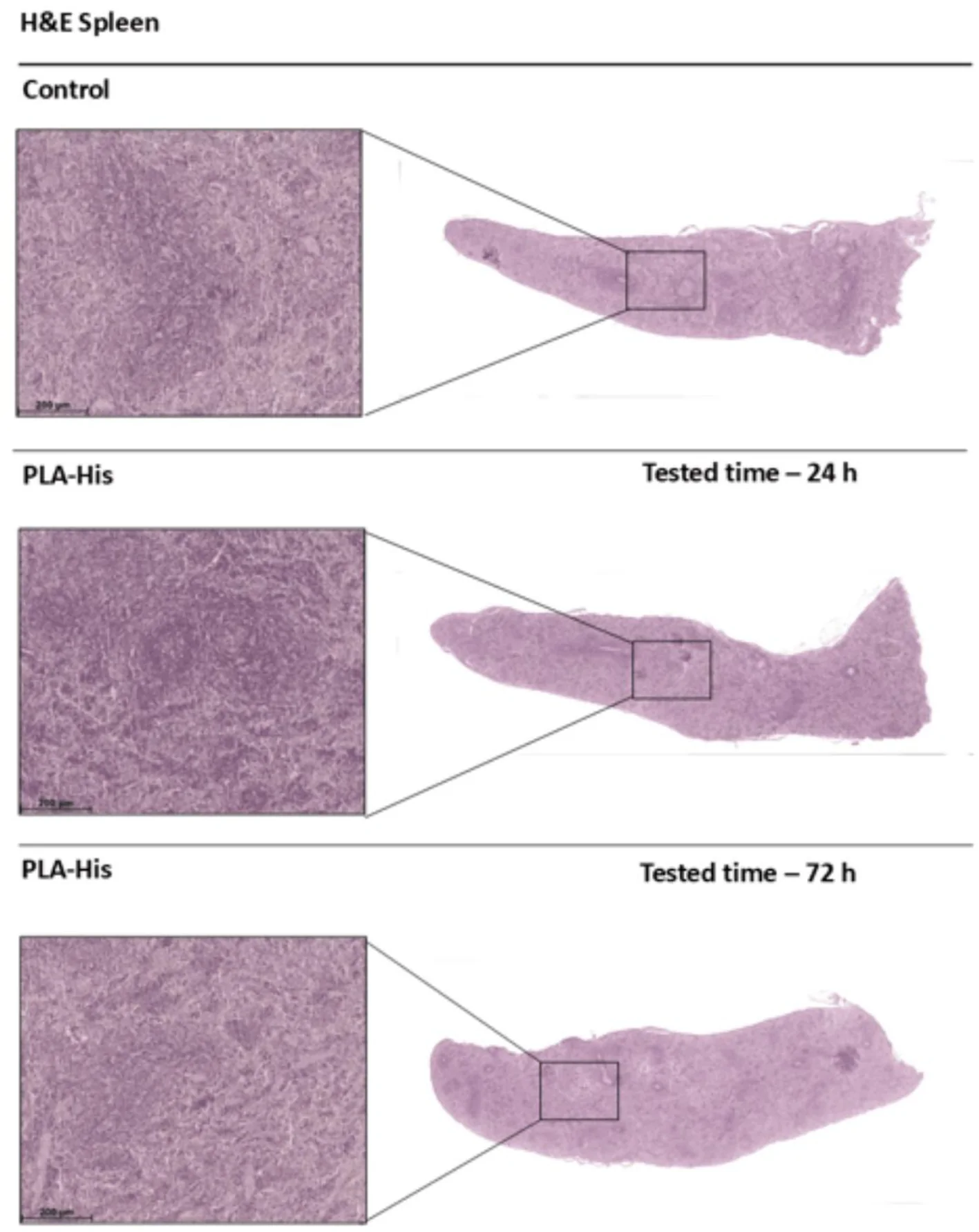
Hematoxylin-eosin (H&E) staining of spleen sections. Animals receiving *per os* tested nanoparticles (NPs) with histamine (His) PLA-His or 0.85% NaCl (control animals). Images were acquired using Leica Mica Microhub (Leica Microsystems, Wetzlar, Germany) at 10x and 40x magnification (water immersion).

**Figure 12:**
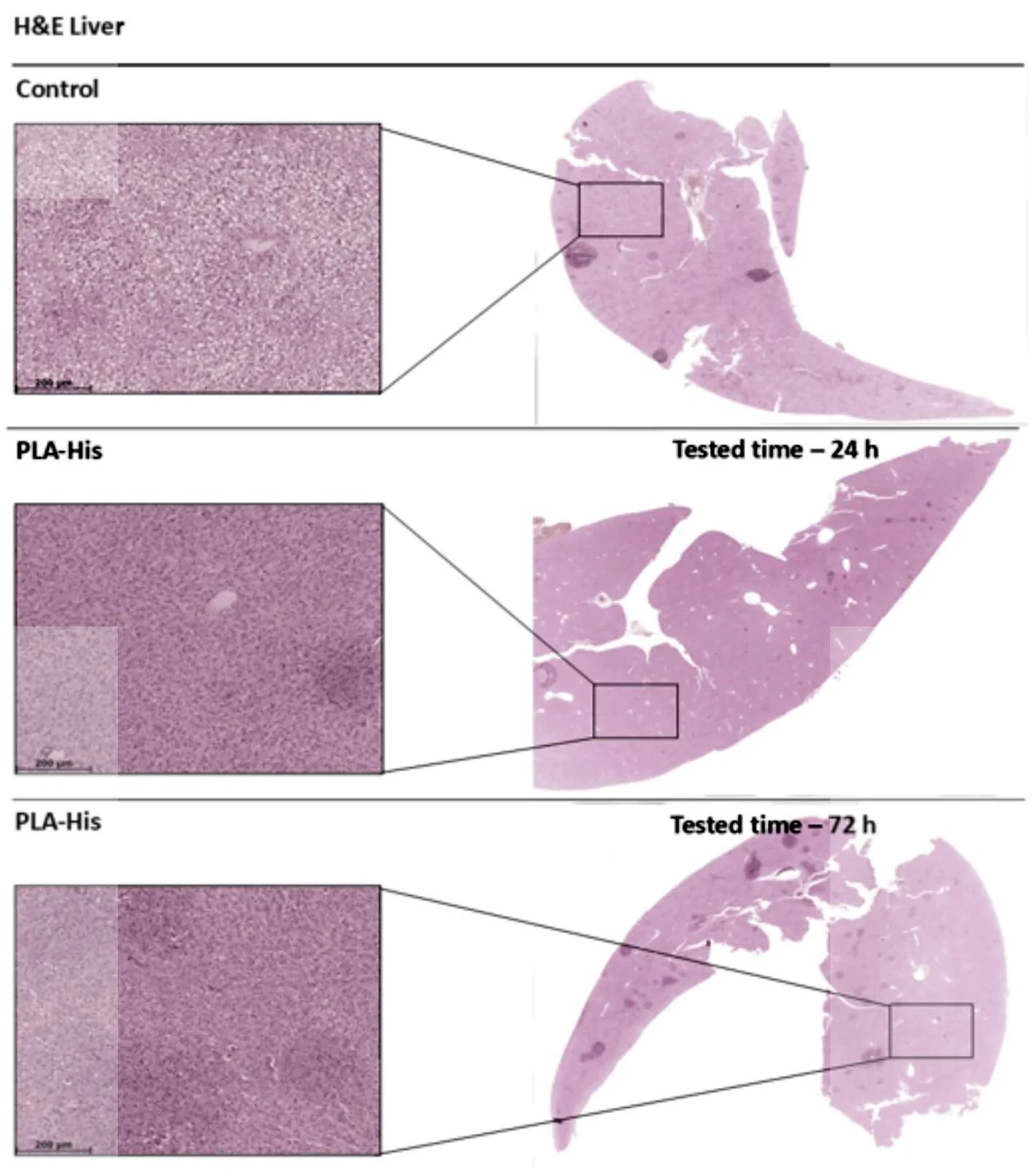
Hematoxylin-eosin (H&E) staining of liver sections. Animals receiving *per os* tested nanoparticles (NPs) with histamine (His) PLA-His or 0.85% NaCl (control animals). Images were acquired using Leica Mica Microhub (Leica Microsystems, Wetzlar, Germany) at 10x and 40x magnification (water immersion).

In this study, guinea pigs were used as *in vivo* model for testing the biocompatibility of empty NPs PLA-His. The systemic and local tissue effects after oral inoculation of animals were examined. Following NPs administration, alanine aminotransferase (ALT) and aspartate aminotransferase (AST) levels in liver tissue homogenates or serum were assessed and they did not differ from assessments in control animals (Figures 9A–D). Moreover, serum levels of CRP, TNF-α, IL-1β, cystatin C, and IgE antibodies were evaluated in animals receiving the NPs tested. These biomarker levels in animals receiving PLA-His NPs were comparable to those in control animals (Figures 9E–I, respectively). H&E staining of liver, kidney and spleen tissue sections revealed no pathological alterations in the NPs-treated animals compared to control animals (Figures 10–12).

## 4 Discussion

Recently, there has been a shift toward developing more physiologically relevant experimental models that more closely reproduce selected features of the tumor microenvironment (TME), including cell–cell and cell–extracellular matrix (ECM) interactions, oxygen gradients, and tumor heterogeneity (Rodrigues et al., 2024; Mangani et al., 2025; Edmondson et al., 2014). 3D GC cell cultures have been recognized as useful experimental models because spheroids reproduce selected structural and functional features of solid tumors, including multicellular organization, spatial gradients, and barriers to drug distribution (Friedrich et al., 2009; Arora et al., 2024). However, spheroids remain simplified and avascular models lacking immune, stromal, and vascular components of the *in vivo* tumor microenvironment. 3D cell models may therefore be suitable for studying nanomaterial-based carriers for anticancer drugs and their distribution within multicellular structures like spheroids (Lazzari et al., 2017).

The current study showed that AGS GC cells reproducibly formed stable multicellular spheroids with a denser central region and a morphologically distinguishable peripheral rim. Because specific spatial markers of proliferation, hypoxia, and necrosis were not used, these regions were identified based on morphology and were not adjusted to definitive biological functions (Arora et al., 2024; Hirschhaeuser et al., 2010).

A novel aspect of this research was the incorporation of soluble *H.pylori* components into a 3D GC model. *H. pylori* are classified as a Group 1 carcinogen and are involved in GC development (Rudnicka et al., 2019). The experimental conditions used in the present study represent *H. pylori* component-conditioned environment rather than active bacterial infection. Soluble recombinant CagA and LPS were treated as extracellular components released from damaged bacterial cells which may induce different biological effects however, they do not reproduce complex conditions during chronic infection. The bacterial components used in the present study, including *H. pylori* CagA, *H. pylori* LPS, and standard *E. coli* LPS, influenced spheroid morphology, particularly the peripheral rim. After 24 h, exposure of spheroids to CagA and LPS resulted in the tendency of enlarging peripheral spheroid rim area. However, the proliferation assay did not demonstrate a corresponding increase in cell proliferative activity. Therefore, rim expansion may reflect changes in cell arrangement, adhesion, cohesion, or spheroid compaction rather than increased migration or proliferation. CagA induced the most pronounced morphological effect among the bacterial components tested. This *H. pylori* component *in vivo* is associated with gastric carcinogenesis and may stimulate proliferation in non-malignant gastric epithelial cells, thereby increasing the probability of accumulating genetic alterations (Covacci et al., 1993; Rokkas et al., 1999). During active infection, CagA is delivered into host cells through the type IV secretion system and interferes with intracellular signaling pathways, cell polarity, and cell–cell adhesion, producing the characteristic elongated or hummingbird phenotype (Segal et al., 1999; Hatakeyama, 2014). Nevertheless, the soluble recombinant CagA used in the present study does not reproduce this physiological mode of delivery. Its effects in the spheroid model should therefore be interpreted as result to extracellular CagA exposure of spheroids rather than as direct equivalent of CagA cell translocation.

Both *H. pylori* and *E. coli* LPS caused transient changes in the spheroid rim during the first 24 h. The mechanism underlying the effect of *H. pylori* LPS remains unclear. In our previous wound-healing study using primary gastric epithelial cells from *Cavia porcellus, H. pylori* LPS, but not *E. coli* LPS, reduced cell migration. This effect was associated with altered collagen I production and interference with pro-regenerative interleukin-33 signaling (Gonciarz et al., 2021). Structural differences between *H. pylori* and *E. coli* LPS may contribute to their distinct biological activities. The apparent differences between the previous primary-cell model and the present AGS spheroid model may also reflect cancer-associated changes in receptor expression, cell adhesion, and three-dimensional organization. The observed increase in rim area may be consistent with altered cell–cell adhesion, polarity, and compaction because *H. pylori* virulence factors are known to affect E-cadherin/β-catenin signaling and epithelial organization. However, direct migration was not measured in the present study (Hatakeyama, 2014; Shirani et al., 2024). Further studies using direct migration and adhesion assays are required to define how bacterial LPS influence spheroid morphology.

Previous studies have been performed on classical model of adherent AGS cells exposed to soluble His, soluble DOX, His combined with DOX, blank PLA and PLA-HisNPs, non-histamine-functionalized PLA-DOX, and PLA-His-DOX (Brzeziński et al., 2023; Jaroniek et al., 2025). These studies revealed the strongest cytotoxic effect toward these cells induced by PLA-His-DOX therefore prompted further investigation using the above formulation and 3D model of AGS cells.

The present study showed better distribution of DOX-associated fluorescence in spheroids treated with PLA-His-DOX than in those exposed to free DOX or PLA-DOX (Figure 1). Because the polymeric carrier was not independently labeled, the fluorescence signal did not distinguish intact NPs from DOX released from the studied formulation. The results therefore indicate enhanced distribution of nanoparticle-delivered DOX rather than direct penetration of intact PLA-His-DOX NPs.

Treatment of spheroids with DOX or PLA-His-DOX for 24 h resulted in increased compaction of the central region and changes in the peripheral rim. These morphological changes were observed together with reduced cell metabolic activity and may therefore be associated with treatment-induced cellular stress. However, spheroid compaction alone does not provide direct evidence of structural collapse or a specific mechanism of cytotoxicity. After 72 h, changes in central compaction and peripheral morphology were observed in cultures exposed to bacterial components, DOX, PLA-His-DOX, or their combinations. Blank PLA-His and PLA-DOX were not included in every functional assay performed in the present study. Therefore, data demonstrate the biological activity of the selected PLA-His-DOX formulation in AGS spheroids but do not independently quantify the contribution of His functionalization to each endpoint under 3D conditions.

No significant increase in cell proliferation or occludin-associated fluorescence was detected in spheroids exposed to bacterial components, DOX, or PLA-His-DOX, whereas ZO-1-associated fluorescence increased in cultures treated with DOX or PLA-His-DOX. The relationship between increased ZO-1-associated fluorescence and spheroid morphology, therefore, remains uncertain. Representative spatial imaging and functional permeability assays are needed to determine whether the observed changes affect intercellular junction organization.

The absence of increased cell proliferation indicates that the time-dependent changes in spheroid morphology cannot be explained by enhanced cell proliferative activity. Similar spatial rearrangements without substantial changes in proliferation indices have been described in other three-dimensional models (Vinci et al., 2012). To further characterize the biological response, metabolic activity was assessed together with oxidative stress, DNA damage, and apoptosis-associated endpoints.

DOX and PLA-His-DOX significantly reduced the metabolic activity of spheroidal cells at 24- and 72-h cultures, whereas bacterial components reduced it mainly after 72 h. The cytotoxic activity of DOX and PLA-His-DOX was maintained in cultures additionally exposed to bacterial components and was particularly evident after 24 h.

Increased cell cytotoxicity in combinatory variants of cell cultures with bacterial components can be interpreted because of bacterial component-induced change in cell organization or junctional integrity, which altered the intratumoral distribution of DOX. However, further study is needed to assess the influence of *H. pylori* components on spheroid permeability or penetration of intact NPs.

Previous reports showed that *H. pylori* virulence factors, including CagA and LPS, may disrupt epithelial organization and promote tissue remodeling (Amieva et al., 2003; Chmiela et al., 2017). Disruption of the gastric epithelial barrier has also been demonstrated in cell cultures *in vitro* and in *Cavia porcellus* infected with *H. pylori* (Mnich et al., 2016; Gonciarz et al., 2019b). Nevertheless, these earlier findings must be confirmed using 3D cell model.

The cytotoxic activity of PLA-His-DOX alone or in combination with bacterial components was associated with increased 4-hydroxynonenal-associated fluorescence, DNA damage, and apoptosis-related responses as shown by the terminal deoxynucleotidyl transferase dUTP nick end labeling assay providing evidence of apoptosis-associated DNA fragmentation or increased cleaved caspase 9.

*H. pylori* CagA and LPS contribute to inflammation and oxidative stress during infection (Kusters et al., 2006; Salvatori et al., 2023; Gonciarz et al., 2019a). In the present study, the cell exposure to bacterial components was associated with increasing of 4-HNE, lipid peroxidation product. However, data interpretation is limited since oxidative stress environment during chronic *H. pylori* infection is more complex.

The pro-apoptotic response was more pronounced in PLA-His-DOX milieu with bacterial components than in cultures exposed to bacterial components alone. These findings are related to enhanced cytotoxic activity of PLA-His-DOX in bacterial component-conditioned spheroid model. Further research is needed to assess whether oxidative stress specifically enhances the therapeutic effectiveness of the studied formulation.

Gastric epithelial cells exposed *in vivo* or *in vitro* to *H. pylori* components may produce inflammatory mediators, including IL-1β, IL-6, and IL-8, and contribute to extracellular matrix degradation through matrix metalloproteinases (Crabtree et al., 1994; Kitadai et al., 2000; Mnich et al., 2015). GC cells may also contribute to development of inflammatory tumor environment (Wen et al., 2022).

In the present study, cell exposure to bacterial components alone did not significantly increase IL-1β or IL-8 concentrations. Lower cytokine concentrations were observed in some cultures combining bacterial components with DOX or PLA-His-DOX compared to respective bacterial components alone. However, PLA-His-DOX reduced cell metabolic activity and increased apoptosis-associated responses. Therefore, because cytokine concentrations were not normalized to viable cell number, DNA content, or total cellular protein, the observed reduction of cytokines may partly reflect the lower number of viable cells or diminished cell metabolic activity. Further research, including assessment of broader panel of inflammatory cytokines (IL-6, tumor necrosis factor-α, monocyte chemoattractant protein-1), and nuclear factor kappa B (NF-κB) signaling is necessary to make conclusions on possible immunomodulatory effects of the studied NPs.

The *in vivo* experiments provided an exploratory short-term assessment of blank PLA-His nanoparticle tolerability in *Cavia porcellus*. No significant changes were observed in the activity of serum or liver alanine aminotransferase and aspartate aminotransferase, serum levels of inflammatory markers, CRP cystatin C, or IgE immunoglobulin. Histological examination did not reveal detectable abnormalities in the liver, kidney, or spleen tissues within 24 and 72 h after oral administration of the studied NPs. These findings provide preliminary evidence of short-term tolerability of the blank carrier under the tested conditions but do not confirm its long-term safety, safety of the DOX-loaded formulation, or readiness for clinical translation. Further research is needed to assess nanoparticle biodistribution and tissue concentrations, pharmacokinetics, gastrointestinal retention, repeated-dose toxicity, or therapeutic efficacy. Gastric and intestinal tissues should be included in additional histological analyses.

In conclusion, the present study using 3D GC cell model confirms and extends the previous findings from conventional cultures of AGS cells. It has been shown that PLA-His-DOX NPs are cytotoxic toward AGS spheroids in the presence or absence of selected soluble *H. pylori* components (CagA and LPS). 3D model is more complex than AGS cell monolayers therefore better mimic tumor environment however, it remains avascular, which is a limitation. The exploratory data obtained in this study prompts deepening anti-cancer mechanisms of the studied NPs by utilizing live bacterial cells, complete nanoparticle control panel in functional *in vitro* assays and further validation of NPs biocompatibility *in vivo*.

### 4.1 Key findings

1. PLA-His-DOX NPs penetrate AGS GC cell spheroids and exert strong cytotoxic effect in 3D conditions.

2. Selected *H. pylori* soluble components (CagA, LPS) modify spheroid architecture by increasing rim layer without promotion of proliferation.

3. PLA-His-DOX NPs show enhanced cytotoxicity toward GC cells under exposure of spheroids to bacterial components suggesting improved delivery of DOX to spheroid milieu.

4. Enhanced cytotoxic effect of the studied PLA-His-DOX NPs can be related to increased oxidative stress, DNA damage, and apoptosis of spheroidal GC cells.

5. PLA-His-DOX NPs diminished the ability of spheroidal cells to deliver pro-inflammatory cytokines.

6. The short-time tolerability of empty PLA-His- NPs was confirmed in guinea pig model based on selected tissue and systemic biomarkers.

This preliminary study presents 3D GC AGS spheroid model facilitating the assessment of tumor microenvironment signatures in response to *H. pylori* components or potentially live bacterial cells and PLA-His-DOX NPs cytotoxicity toward spheroidal GC cells partially mimicking this cell architecture *in vivo*.

### 4.2 Limitations of the study

Although 3D spheroid models better mimic certain structural features of solid tumors than traditional two-dimensional cell cultures, they have remained simplified experimental tumor models. The AGS spheroids used in this study lack vascularization, immune cell infiltration, and dynamic stromal–tumor interactions, all of which may impact tumor organization, inflammatory signaling, NPs transport, and therapeutic response *in vivo*. Central and peripheral regions of the spheroids were distinguished based on morphological appearance without recognition of specific spatial markers of proliferation, hypoxia, and necrosis.

The study employed only a single gastric adenocarcinoma cell line, AGS, therefore the representation of the molecular, histological, and interpatient heterogeneity characteristic of GC is limited. Consequently, findings cannot be generalized to all GC subtypes. Further validation with additional GC cell lines, non-malignant gastric epithelial cells, stromal and immune cell co-cultures, and patient-derived GC organoids would enhance the biological and translational relevance of the model.

The impact of *H. pylori* on spheroid architecture and GC cell activity was simulated using soluble recombinant CagA protein and LPS. Therefore, the results illustrate responses to selected soluble bacterial components released *in vivo* from bacterial cells, but not the comprehensive host–pathogen interactions involved in *H. pylori*-associated gastric carcinogenesis. It does not reflect the prolonged epithelial, stromal, and immune remodeling associated with chronic *H. pylori* colonization.

The PLA-His-DOX formulation was selected based on previous studies using 2D GC cell cultures, in which soluble His and DOX, blank PLA, PLA-His NPs non-histamine-functionalized PLA-DOX, and PLA-His-DOX were comparatively evaluated concerning anti-GC cell activity. The present investigation was designed as a subsequent assessment of this formulation activity within a more complex 3D GC cell model. The current results demonstrate the biological activity of PLA-His-DOX under 3D conditions but do not establish the specific contribution of His functionalization to each measured endpoint.

The assessment of distribution and intracellular fate of the studied NPs is preliminary and reflects the distribution of nanoparticle-delivered DOX, but cannot differentiate between intact NPs and drug released before or after entering the spheroid because carrier was not separately fluorescently labeled. The role of His receptors, individual endocytic pathways, intracellular trafficking, and subcellular drug localization should be further examined.

The analysis of inflammatory cell response was limited to IL-1β and IL-8 therefore, the assessment of broader cytokine panel and NF-kB should be added in a future study following normalization to viable cell number to be able to make conclusion about immunomodulatory effects of the studied NPs. In this study lower cytokine levels in certain spheroid cultures may illustrate the immunomodulatory activity of the studied NPs however, it may partly result from diminished cell viability as shown by MTT reduction assay.

*In vivo* preliminary study showed short-term biocompatibility of blank PLA-His NPs 24 or 72 h after oral administration of this formulation. Further study is necessary to evaluate biocompatibility of DOX-loaded NPs including biodistribution, pharmacokinetics, clearance pathways, gastrointestinal retention, repeated-dose toxicity, or antitumor efficacy. Histological analysis lack gastric or intestinal tissues, therefore conclusions on local tolerability following oral administration of the studied NPs are missing. The relatively small animal groups and absence of a formal prospective power calculation also warrant cautious interpretation. Overall, these findings offer preliminary evidence of the short-term tolerability of the blank carrier under the tested conditions but do not establish long-term biocompatibility of the carrier. Moreover, PLA-His-DOX therapeutic efficacy or readiness for clinical translation should be established.

## 5 Conclusions

This study established a 3D culture model of AGS GC cell (spheroid model) mimicking tumor-like cell architecture. This model facilitated to assess the influence of selected soluble *H. pylori* components, CagA and LPS, on spheroidal architecture and the activity of PLA-His-DOX NPs toward spheroid GC cells. Since soluble bacterial components were used, the model reflects an environment conditioned by *H. pylori* components and does not replicate infection with live bacterial cells. Cell exposure to *H. pylori* CagA or LPS influenced spheroid morphology, increasing the outer rim area. These structural alterations were not related to increased cell proliferation indicating rather changes in cell arrangement, adhesion, cohesion, or compaction. PLA-His-DOX NPs facilitated distribution of released DOX within AGS spheroids and decreased the metabolic activity of spheroidal cells. Its biological effects included increased lipid peroxidation, DNA damage, and apoptosis-related responses. Lower levels of IL-1β and IL-8 which were shown in certain cell cultures containing bacterial components and DOX or PLA-His-DOX may reflect anti-inflammatory effect of PLA-His-DOX. These findings should be interpreted with caution because bacterial components alone did not significantly boost cytokine production, and cytokine levels were not normalized to cell viability, DNA content, or total cellular protein. Thus, the reduction of cytokines may partly result from decreased cell viability and do not independently confirm a specific anti-inflammatory effect of PLA-His-DOX.

*In vivo* study demonstrated a short-term tolerability of blank PLA-His after oral administration to guinea pigs. Further research is needed to assess biodistribution, long-term safety, gastrointestinal tolerability of blank carrier *in vivo* as well as biocompatibility and antitumor efficacy of PLA-His-DOX. Overall, the developed AGS spheroid model offers a valuable proof-of-concept platform for investigating the impact of *H. pylori* on GC cell organization and for evaluating the activity of nanocarrier-delivered anticancer drugs. Future research should incorporate additional GC cell models, live *H. pylori*, independently labeled NPs, with adjacent controls, expanded inflammatory analyses, and appropriate *in vivo* GC models to determine pharmacokinetics, tissue distribution, tolerability, and therapeutic efficacy of PLA-His-DOX NPs.

## Data Availability

The raw data supporting the conclusions of this article will be made available by the authors, without undue reservation.
