# Supplementary material for: Development of 3D spheroids of AGS gastric cancer cells in vitro for exploration of H. pylori influence on tumor environment and anti-cancer activity of PLA-His-DOX nanoparticles
Source: Front Microbiol. 2026 Aug 24;17:1935725. doi: 10.3389/fmicb.2026.1935725 (PMC13547500; doi:10.3389/fmicb.2026.1935725)
Supplement: Supplementary file 1 [file Data_Sheet_1.docx]

# Supplementary Material

**Supplementary Table S1.** Physicochemical characteristics of the newly prepared PLA-His and PLA-His-DOX nanoparticle formulations and nominal composition of PLA-His-DOX used in biological experiments.

| Parameter | PLA-His | PLA-His-DOX |
| --- | --- | --- |
| Mean hydrodynamic diameter | 67 nm | 78 nm |
| Polydispersity index | 0.236 | 0.184 |
| Zeta potential | −16.0 mV | −16.8 mV |
| DOX encapsulation efficiency | Not applicable | 49% |
| Calculated encapsulated DOX | Not applicable | 0.735 mg |
| Nominal drug loading | Not applicable | 4.99% w/w |
| Nominal total NP concentration at 1 µg/mL DOX | Not applicable | 20.0 µg/mL |
| Nominal total polymer concentration | Not applicable | 19.0 µg/mL |
| Nominal PLA-His concentration | Not applicable | 13.6 µg/mL |
| Nominal PEGME-*b*-PDLLA concentration | Not applicable | 5.4 µg/mL |

Drug loading and nanoparticle/polymer concentrations were calculated from the formulation composition and encapsulation efficiency and were not determined directly by gravimetric analysis.

## Supplementary Figures

Supplementary Material

**Supplementary Figure S1. Representative physicochemical characterization of the newly prepared PLA-His and PLA-His-DOX nanoparticles.** Representative particle-size distributions determined by dynamic light scattering are shown for (A) blank PLA-His and (B) PLA-His-DOX nanoparticles. Representative zeta-potential distributions determined by electrophoretic light scattering are shown for (C) blank PLA-His and (D) PLA-His-DOX nanoparticles. The mean hydrodynamic diameters were 67 and 78 nm, the polydispersity index values were 0.236 and 0.184, and the zeta potentials were −16.0 and −16.8 mV for PLA-His and PLA-His-DOX, respectively.

**Supplementary Figure** 2**. Total projected area of spheroids after 24 and 72 h of exposure to bacterial components, DOX, or PLA-His-DOX.** The total two-dimensional area enclosed by the spheroid contour was measured from spatially calibrated transmitted-light images using ImageJ software. Data are presented as mean ± standard error of the mean (SEM) from three independent experiments (*n* = 3). In each experiment, four technical replicates were analyzed for each condition and averaged before statistical analysis. Comparisons between two groups were performed using the Mann–Whitney U test. Comparisons involving more than two groups were performed using the Kruskal–Wallis test followed by Dunn’s post hoc test. Differences were considered statistically significant at *p* < 0.05. * indicates medium versus cells exposed to bacterial components, DOX, PLA-His-DOX, or their combinations; ■ indicates a bacterial component alone versus the corresponding bacterial component combined with DOX or PLA-His-DOX. CagA, cytotoxin-associated gene A protein; LPS Hp, *Helicobacter pylori* lipopolysaccharide; LPS Ec, *Escherichia coli* lipopolysaccharide; DOX, doxorubicin; PLA-His-DOX, histamine-functionalized polylactide nanoparticles loaded with DOX.

*p < 0.05, **p < 0.01, ***p < 0.001.

Supplementary Material

**Supplementary Figure 3. Diameter of AGS spheroids after 24 and 72 h of exposure to bacterial components, DOX, PLA-His-DOX, or their combinations.** Spheroid diameter was measured from spatially calibrated transmitted-light images using ImageJ software and expressed in micrometers. Data are presented as mean ± standard error of the mean (SEM) from three independent experiments (*n* = 3). In each experiment, four technical replicates were analyzed for each condition and averaged before statistical analysis. Comparisons between two groups were performed using the Mann–Whitney U test. Comparisons involving more than two groups were performed using the Kruskal–Wallis test followed by Dunn’s post hoc test. Differences were considered statistically significant at *p* < 0.05. * indicates a comparison between spheroids maintained in medium and spheroids exposed to bacterial components, DOX, PLA-His-DOX, or their combinations; ■ indicates a comparison between a bacterial component alone and the corresponding bacterial component combined with DOX or PLA-His-DOX. CagA, cytotoxin-associated gene A protein; LPS Hp, *Helicobacter pylori* lipopolysaccharide; LPS Ec, *Escherichia coli* lipopolysaccharide; DOX, doxorubicin; PLA-His-DOX, histamine-functionalized polylactide nanoparticles loaded with doxorubicin. *p < 0.05, **p < 0.01, ***p < 0.001.

**Supplementary Figure 4. Circularity of AGS spheroids after 24 and 72 h of exposure to bacterial components, DOX, PLA-His-DOX, or their combinations.** Circularity was determined from the spheroid contours using ImageJ software according to the formula 4π × area/perimeter².

Circularity values range from 0 to 1, with values approaching 1 indicating a more circular and regular spheroid outline. Data are presented as mean ± standard error of the mean (SEM) from three independent experiments (*n* = 3). In each experiment, four technical replicates were analyzed for each condition and averaged before statistical analysis. Comparisons between two groups were performed using the Mann–Whitney U test. Comparisons involving more than two groups were performed using the Kruskal–Wallis test followed by Dunn’s post hoc test. Differences were considered statistically significant at *p* < 0.05. * indicates a comparison between spheroids maintained in medium and spheroids exposed to bacterial components, DOX, PLA-His-DOX, or their combinations; ■ indicates a comparison between a bacterial component alone and the corresponding bacterial component combined with DOX or PLA-His-DOX. CagA, cytotoxin-associated gene A protein; LPS Hp, *Helicobacter pylori* lipopolysaccharide; LPS Ec, *Escherichia coli* lipopolysaccharide; DOX, doxorubicin; PLA-His-DOX, histamine-functionalized polylactide nanoparticles loaded with doxorubicin. *p < 0.05, **p < 0.01, ***p < 0.001.

Supplementary Material

**Supplementary Figure 5.** The influence of bacterial components and DOX or PLA-His-DOX on spheroid ZO-1 (A), occludin (B) production and proliferative activity (C). The median values were assessed using the U Mann-Whitney test or Kruskal-Wallis test, followed by Dunn’s post-hoc test with a significance level of p < 0.05. * cells in medium vs cells stimulated. CagA, cytotoxin-associated gene A; LPS Hp, *H. pylori* lipopolisaccharide; LPS Ec, *E. coli* lipopolisaccharide DOX, doxorubicin; PLA-His-DOX, NPs PLA modified with histamine (His) and loaded with DOX. Data are presented as mean

± SEM from three independent experiments (*n* = 3). Four technical replicates were analyzed for each condition in each experiment and averaged before statistical analysis.*p < 0.05, **p < 0.01, ***p < 0.001.

**Supplementary Figure 6.** The influence of bacterial components and DOX or PLA-His-DOX on IL-1β in AGS spheroid culture supernatants. Spheroids were exposed for 24 or 72 h to CagA, *Helicobacter pylori* lipopolysaccharide (LPS Hp), *Escherichia coli* lipopolysaccharide (LPS Ec), doxorubicin (DOX), or histamine-functionalized polylactide nanoparticles loaded with doxorubicin (PLA-His-DOX), either alone or in the indicated combinations. The median values were compared using the Mann–Whitney *U* test or the Kruskal–Wallis test followed by Dunn’s post hoc test, as appropriate. Statistical significance was set at *p* < 0.05. * indicates cells maintained in medium alone versus cells exposed to the indicated treatment; ■ indicates cells exposed to a bacterial component alone versus cells exposed to the corresponding bacterial component combined with DOX or PLA-His-DOX. CagA, cytotoxin-associated gene A protein; LPS Hp, *H. pylori* lipopolysaccharide; LPS Ec, *E. coli* lipopolysaccharide; DOX, doxorubicin; PLA-His-DOX, histamine-functionalized polylactide nanoparticles loaded with doxorubicin. Data are presented as mean ± SEM from three independent experiments (*n* = 3). Four technical replicates were analyzed for each condition in each experiment and averaged before statistical analysis. *p < 0.05, **p < 0.01, ***p < 0 .001.

Supplementary Material

**Supplementary Figure 7.** The influence of bacterial components and DOX or PLA-His-DOX on IL-8 concentrations in AGS spheroid culture supernatants. Spheroids were exposed for 24 or 72 h to CagA, *Helicobacter pylori* lipopolysaccharide (LPS Hp), *Escherichia coli* lipopolysaccharide (LPS Ec), doxorubicin (DOX), or histamine-functionalized polylactide nanoparticles loaded with doxorubicin (PLA-His-DOX), either alone or in the indicated combinations. The median values were compared using the Mann–Whitney *U* test or the Kruskal–Wallis test followed by Dunn’s post hoc test, as appropriate. Statistical significance was set at *p* < 0.05. * indicates cells maintained in medium alone versus cells exposed to the indicated treatment; ■ indicates cells exposed to a bacterial component alone versus cells exposed to the corresponding bacterial component combined with DOX or PLA-His-DOX. CagA, cytotoxin-associated gene A protein; LPS Hp, *H. pylori* lipopolysaccharide; LPS Ec, *E. coli* lipopolysaccharide; DOX, doxorubicin; PLA-His-DOX, histamine-functionalized polylactide nanoparticles loaded with doxorubicin. Data are presented as mean ± SEM from three independent experiments (*n* = 3). Four technical replicates were analyzed for each condition in each experiment and averaged before statistical analysis.*p < 0.05, **p < 0.01, ***p < 0.001.
